# Endothelial Myosin IIA Is Required for the Maintenance of Blood–Brain Barrier Integrity

**DOI:** 10.3390/cells13191635

**Published:** 2024-10-01

**Authors:** Yanan Deng, Ziqi Qiao, Changping Zhou, Yujun Pei, Han Xu, Xuya Kang, Jincai Luo

**Affiliations:** 1 Beijing Key Laboratory of Cardiometabolic Molecular Medicine, Institute of Molecular Medicine, School of Future Technology, Peking University, Beijing 100871, China; 2 College of Future Technology, Peking University, Beijing 100871, China

**Keywords:** blood–brain barrier, endothelium, animal models, vascular biology, adhesion molecules

## Abstract

Brain endothelial cells (ECs) are essential elements of the blood–brain barrier (BBB), maintaining its integrity through both paracellular junctions and transcellular transport systems. Myosin IIA, a multifunctional protein, plays a significant role in various cellular processes, including cytoskeletal maintenance, cell division, and signal transduction. While Myosin IIA has been implicated in bleeding and ischemic stroke, its role in regulating BBB integrity under physiological conditions remains unclear. In this study, we investigated the impact of Myosin IIA deficiency on BBB integrity using intravenous tracer injections and models of epilepsy. Flow cytometry, Western blot, and real-time PCR were employed to isolate brain cells and assess changes in protein and mRNA levels. Additionally, immunofluorescence staining and electron microscopy were used to explore alterations in protein expression and the structure of BBB. Our results demonstrate that endothelial Myosin IIA deficiency increased BBB permeability and exacerbated symptoms in BBB-related diseases. Mechanistically, we found that Myosin IIA modulates β-catenin transcription and protein interactions. The overexpression of β-catenin in brain endothelial Myosin IIA deficiency mice improved BBB integrity and reduced disease severity. This study establishes Myosin IIA as a critical regulator of BBB integrity and suggests new therapeutic targets for vascular diseases.

## 1. Introduction

The blood–brain barrier (BBB) plays a pivotal role in safeguarding the brain against foreign pathogens and harmful substances of blood origin [1,2]. Brain endothelial cells (ECs) are crucial components of the BBB and exert their influence through paracellular connections and transcellular transport mechanisms [3,4,5]. The former phenomenon can be attributed to tight junction complexes, which are composed of tight junction proteins, such as claudins and occludin, and adhesion molecules like VE-cadherin and β-catenin, within the brain ECs. The latter is due to the extremely low proportion of transcytosis in brain ECs. Additionally, these ECs express transporters like glucose transporters (Glut1), which are responsible for facilitating the bidirectional transport of essential nutrients to and from the brain [6,7]. Furthermore, transcellular transport mechanisms are not limited to the transport of nutrients but also include various transport systems that modulate the blood-to-brain transport of drugs and other solutes. Organic anion-transporting polypeptides and organic cation transporters play significant roles in mediating the uptake of drugs and other solutes into the brain. On the other hand, efflux transporters such as P-glycoprotein, breast cancer resistance protein, and multidrug resistance-associated proteins are crucial in restricting the transcellular permeability of circulating solutes, thereby protecting the brain from potentially harmful substances [8,9]. In addition, interactions between the endothelium and other brain cells, such as pericytes and astrocytes, significantly contribute to regulating BBB integrity [10,11,12,13,14]. BBB permeability increases progressively during aging [15,16], and BBB breakdown is indeed a hallmark feature reported in numerous brain diseases [17,18,19]. BBB injury can cause or exacerbate neuronal dysfunction and many CNS disorders, including brain tumors, epilepsy, multiple sclerosis, and Alzheimer’s disease [20,21,22,23]. Recent studies have reported an effective therapeutic approach to modulate BBB function by enhancing endogenous developmental signaling [24]. However, there remains a need for additional research to explore the targets and mechanisms that underlie the regulation of the BBB, especially under physiological status.

Myosin IIA is an adhesion molecule with multiple functions [25]. Previously, we highlighted Myosin IIA as an important regulator of endothelial von Willebrand Factor secretion, which contributes to endothelial coagulation and vascular homeostasis against injury [26,27,28]. On the other hand, there has been an interest in the function of Myosin IIA on cerebral microvasculature. An early study showed that Myosin IIA-deficient mice exhibit embryonic lethality characterized by significant defects in brain vasculature due to the abnormal development of placental blood vessels [29,30]. A recent study demonstrated that Myosin IIA expression in the brain endothelium is altered in mice after ischemia-reperfusion and may play a causative role in CNS injury [31]. BBB is a crucial component of the neurovascular unit (NVU), which consists of endothelial cells, pericytes, astrocytes, neurons, and the extracellular matrix. The NVU functions as an integrated system, coordinating various cellular interactions to maintain the homeostasis of the CNS and regulate BBB permeability [32,33]. Recent research emphasizes the importance of the NVU in modulating BBB integrity, particularly through the interactions between endothelial cells and other components such as pericytes and astrocytes [34]. Understanding the dynamic relationship between the components of the NVU, including how endothelial Myosin IIA interacts within this unit, is essential for uncovering mechanisms underlying BBB regulation under physiological conditions.

While the BBB plays a well-established role in maintaining central nervous system homeostasis, the specific involvement of Myosin IIA in regulating BBB integrity under physiological conditions remains insufficiently understood. To address this, we generated mice with brain endothelial cell-specific Myosin IIA deficiency. Our findings demonstrate that endothelial Myosin IIA deficiency under physiological conditions leads to increased BBB permeability and exacerbates the progression of BBB-related diseases, such as epilepsy. Mechanistically, we identified that Myosin IIA modulates β-catenin transcription and protein interactions and that β-catenin overexpression in Myosin IIA-deficient mice effectively restored BBB integrity and alleviated disease severity. These results highlight the critical role of Myosin IIA in maintaining BBB integrity under physiological conditions and suggest that targeting the Myosin IIA-β-catenin pathway may offer novel therapeutic strategies for vascular and neurological diseases.

## 2. Materials and Methods

### 2.1. Animals

Mice carrying *Myh9* floxed alleles, sourced from Jackson Laboratories, *SP-A*-Cre, and *Cdh5*-CreER transgenic mice, were used in this study. Tamoxifen was used to induce *Cdh5*-CreER recombinase activity in *Myh9* floxed mice, leading to the conditional knockdown of Myosin IIA in endothelial cells. The mice used in this study were all male. They were housed at the Center for Experimental Animals, Peking University, Beijing, China, a facility accredited by the Association for Assessment and Accreditation of Laboratory Animal Care. The animals were maintained in a controlled environment with a 12 h light/12 h dark cycle and had ad libitum access to standard rodent chow and water. The animal experiments in this study were conducted in adherence to the 3R’s principles (Replacement, Reduction, and Refinement) to ensure the ethical use of animals. All animal-related procedures adhered to protocols approved by the Committee for Animal Research of Peking University and complied with the Guide for the Care and Use of Laboratory Animals. Every effort was made to minimize animal suffering and use the minimal number of animals necessary for reliable results.

### 2.2. Cell Lines

Human embryonic kidney (HEK293T) cells were cultured at 37 °C in a humidified atmosphere with 5% CO_2_. The culture medium consisted of Dulbecco’s Modified Eagle’s Medium (DMEM, C11995500BT, Invitrogen, Carlsbad, CA, USA) supplemented with 10% fetal bovine serum (FBS). The mouse and human brain EC line, bEnd.3 (CRL-2299, ATCC, Manassas, DC, USA) and HCMEC/D3, was cultured at 37 °C in a humidified atmosphere with 5% CO_2_. The culture medium consisted of DMEM supplemented with 10% FBS (10091148, Life Technologies, Shanghai, China) and 1% antibiotic–antimycotic (15240062, Life Technologies, Shanghai, China). To ensure the stability of the experimental results, all cell lines used in this study did not exceed passage 20 [35,36,37].

### 2.3. FACS Sorting and Analysis of Brain ECs

Brains were harvested from 8-week-old *Myh9*^fl/fl^ and *Myh9*^ECKO^ mice to isolate primary brain ECs. The brain tissue was dissected and digested in 1% collagenase II (17101015, Gibco, Waltham, MA, USA) at 37 °C for 30 min. Following digestion, the cell pellets were resuspended in 20% bovine serum albumin (BSA) and centrifuged at 1000× *g* at 4 °C for 20 min. Subsequently, the cell pellets were resuspended in 0.5% BSA/phosphate-buffered saline (PBS). The isolated cells were labeled with the following antibodies for 30 min at 4 °C: FITC-conjugated anti-rat CD31 (11-0311-85, eBiosciences, San Diego, CA, USA), Per-cp-conjugated anti-mouse CD45 (45-0415-82, eBiosciences, San Diego, CA, USA), PE-conjugated anti-mouse Pdgfrβ (12-1402-81, eBiosciences, San Diego, CA, USA), and BV506-conjugated fixable viability dye (65-0866-14, eBiosciences, San Diego, CA, USA). After two washes, the cell pellets were resuspended in 0.5% BSA/FBS. FITC-CD31^+^/PE-Pdgfrβ^−^/Per-cp-CD45^−^/BV506^−^ cells were sorted using an Arial III Sorter (BD). The sorted cells were centrifuged at 2000× *g* at 4 °C.

### 2.4. Evans Blue Leakage

Evans blue dye (2.5% in saline, 4 mL/kg body weight, E2129-50G, Sigma Aldrich, Beijing, China) was administered intravenously to P60 mice. Following dye injection, the mice were transcardially perfused with saline. Subsequently, the brains were carefully dissected, weighed, and photographed. Evans blue dye was extracted with formamide (W610419. Energy, Beijing, China) for 48 h at 60 °C. The samples were centrifugated at 3000× *g* for 20 min to separate cellular debris and obtain supernatants. The resulting supernatants were assessed for optical density at 620 nm using a spectrometer (Multiskan Spectrum, Thermo Fisher, Waltham, MA, USA).

### 2.5. BBB Permeability Assay

Mice aged 10–12 weeks received intravenous injections of EZ-Link™ Sulfo-NHS-Biotin (0.2 mg/g; 21335, Invitrogen, Carlsbad, CA, USA), which is dissolved in saline. The solution was freshly prepared before each experiment to ensure its stability and efficacy. Based on the descriptions from the published literature, Sulfo-NHS-Biotin was allowed to circulate for 5 min after injection [38,39]. For the experimental group injected with Sulfo-NHS-Biotin, after the tracers had circulated for a certain period, the mice were euthanized and then transcardially perfused with saline. Following perfusion, brains were carefully removed, fixed overnight in 4% paraformaldehyde (PFA), and equilibrated in 30% sucrose. Floating coronal serial sections 40 μm thick throughout the dentate gyrus were maintained sequentially. The sections were blocked with TBS++ (containing 3% donkey serum and 0.3% Triton X-100) for 1 h at room temperature (RT). Subsequently, they were stained with Streptavidin, Alexa Fluor™ 488 conjugate (S11223, 1:500, Thermo Fisher, Waltham, MA, USA) for 2 hat RT or anti-CD31 (goat, AF3628, R&D, Shanghai, China) overnight at 4 °C with primary antibodies, after primary antibody incubation, the sections were incubated with the corresponding Alexa Fluor-conjugated secondary antibodies (1:1000, Thermo Fisher, Waltham, MA, USA).,

### 2.6. Immunohistochemistry

Mice were euthanized and then transcardially perfused with saline. Following perfusion, brains were carefully removed, fixed overnight in 4% PFA, and equilibrated in 30% sucrose. Floating coronal serial sections 40 μm thick throughout the dentate gyrus were maintained sequentially. The sections were blocked with TBS++ (containing 3% donkey serum and 0.3% Triton X-100) for 1 h at RT. Subsequently, they were stained overnight at 4 °C with primary antibodies. The following antibodies were used: anti-Claudin-5 (rabbit, 341600, Invitrogen, Carlsbad, CA, USA), anti-Occludin (rabbit, 711500, Invitrogen, Carlsbad, CA, USA), anti-Zo-1 (rabbit, 402200, Invitrogen, Carlsbad, CA, USA), anti-Myosin IIA (rabbit, 14844-1-AP, Proteintech, Wuhan, China), anti-β-catenin (rabbit, 8480T, Cell Signaling Technology), anti-CD31 (goat, AF3628, R&D, Shanghai, China), anti-GFAP (rabbit, AB5804, Sigma Aldrich, Beijing, China), anti-Pdgfrβ (rat, 14-1402-82, Invitrogen, Carlsbad, CA, USA), anti-Laminin (rabbit, L9393, Sigma Aldrich, Beijing, China), and anti-Glut1 (rabbit, ab652, Abcam, Shanghai, China). All primary antibodies used are listed in Table S1. After primary antibody incubation, the sections were incubated with the corresponding Alexa Fluor-conjugated secondary antibodies (1:1000, Thermo Fisher, Waltham, MA, USA). Slides were mounted using Prolong (Invitrogen, Carlsbad, CA, USA) and visualized under a laser scanning confocal microscope (LSM 880 microscope, Carl Zeiss AG, Oberkochen, Germany). Image processing was carried out using ImageJ 1.41 and Adobe Photoshop 2023. Immunohistochemistry experiments were independently repeated at least three times to ensure the reliability of the results.

### 2.7. Transmission Electron Microscopy (TEM)

Mouse brains were meticulously dissected and fixed by immersion in a solution composed of 0.1 M sodium cacodylate buffer, 2% glutaraldehyde, and 4% PFA for 1 h at RT, followed by an additional 12 h in 4 °C. Following fixation, the tissue was rinsed twice in 0.1 M sodium cacodylate buffer and then cut into 50 μm thick free-floating sections on a vibratome. The sections were post-fixed in a solution containing 1% osmium tetroxide and 1.5% potassium ferrocyanide. Then, they were dehydrated and embedded in epoxy resin. As previously described, TEM imaging of sections from P60 mice injected with horseradish peroxidase (HRP) was conducted. P30 mice were anesthetized, and HRP type II (P8250-50KU, Sigma Aldrich, Beijing, China) 0.5 mg/g body weight dissolved in 400 mL PBS was bilaterally injected into the retro-orbital sinus. After 30 min of circulation, the brains were dissected and fixed by immersion in a mixture of 0.1 M sodium cacodylate buffer, 5% glutaraldehyde, and 4% PFA for 1 h at RT, followed by overnight immersion in 4% PFA/0.1 M sodium cacodylate at 4 °C. Subsequently, the brain was cut into 50 μm thick free-floating sections on a vibratome. These sections were then incubated in a 3,3′-diaminobenzidine (DAB) solution (ZLI-9018, ZSGB-BIO, Beijing, China) with 0.01% hydrogen peroxide for 45 min at RT. After DAB staining, the sections were post-fixed in 1% osmium tetroxide and 1.5% potassium ferrocyanide, dehydrated, and embedded in epoxy resin. Ultrathin sections (80 nm thick) were cut from the relevant area, collected on copper grids, stained with lead citrate, and examined using a 120 kV TEM.

### 2.8. Western Blot

Brain ECs and cell precipitates were lysed in radioimmunoprecipitation assay (RIPA, R0020, Solarbio, Beijing, China), buffer-supplemented with PMSF protease inhibitor (P0100, Solarbio, Beijing, China). Using standard Western blot techniques, the lysates were fractionated on a 10% SDS-polyacrylamide gel. All primary and secondary antibodies used are listed in Table S2.

### 2.9. Real-Time PCR (RT-PCR) Analysis

The total RNA was extracted from the brain ECs using the RNeasy Mini Kit (DP420, Tiangen, Beijing, China), following the manufacturer’s instructions. Reverse transcription was carried out using the TransScript-One-Step gDNA Removal and cDNA Synthesis SuperMix Kit (AT311-03, Transgene, Beijing, China) with oligo dt primers. RT-PCR assays were performed on a real-time (Biorat) system using PerfectStartTM Green qPCR SuperMix (AQ601-04, Transgene, Beijing, China) as the detection method. We used the comparative Ct method to analyze RNA expression, normalizing it to *GAPDH* as a reference gene. The sequences of primers for RT-PCR are listed in Table S3.

### 2.10. Seizure Model

Male mice aged 6-8 weeks, weighing 18–25 g, received scopolamine (10 mg/kg; S866852, Macklin, Shanghai, China) dissolved in 0.9% saline via intraperitoneal (i.p.) injection. After 30 min, mice were administered pilocarpine hydrochloride (300 mg/kg; HY-B0726, Medchem Express, Beijing, China) dissolved in 0.9% saline via i.p. injection. Following pilocarpine administration, the mice were closely monitored for 120 min. During this period, their behavior was assessed using the Racine scale to identify seizure signs. The surviving mice were euthanized 120 min after pilocarpine administration using an overdose of isoflurane. Only animals displaying seizure signs according to the Racine scale criteria were included in the study. The timing of seizure activity involving rearing and falling and overall survival up to 120 min post-pilocarpine administration was recorded.

### 2.11. Statistics

All experiments were repeated in at least 3 independent experiments. Data are expressed as the mean ± SD. GraphPad Prism Version 9 (GraphPad Software, San Diego, CA, USA) was used for statistical analysis. Differences between the two groups were analyzed by a Student’s *t*-test after the demonstration of homogeneity of variance with an F test. Statistical analyses were conducted with a Student’s *t*-test, Fisher’s exact test, the log-rank (Mantel–Cox) test, and two-way ANOVA. The statistical tests used are indicated in each figure legend. * *p* < 0.05, ** *p* < 0.01, and *** *p* < 0.001 were considered statistically significant.

## 3. Results

### 3.1. Cerebral Endothelial Deletion of Myosin IIA Impairs the Integrity of the BBB in Mice

**Figure 1:**
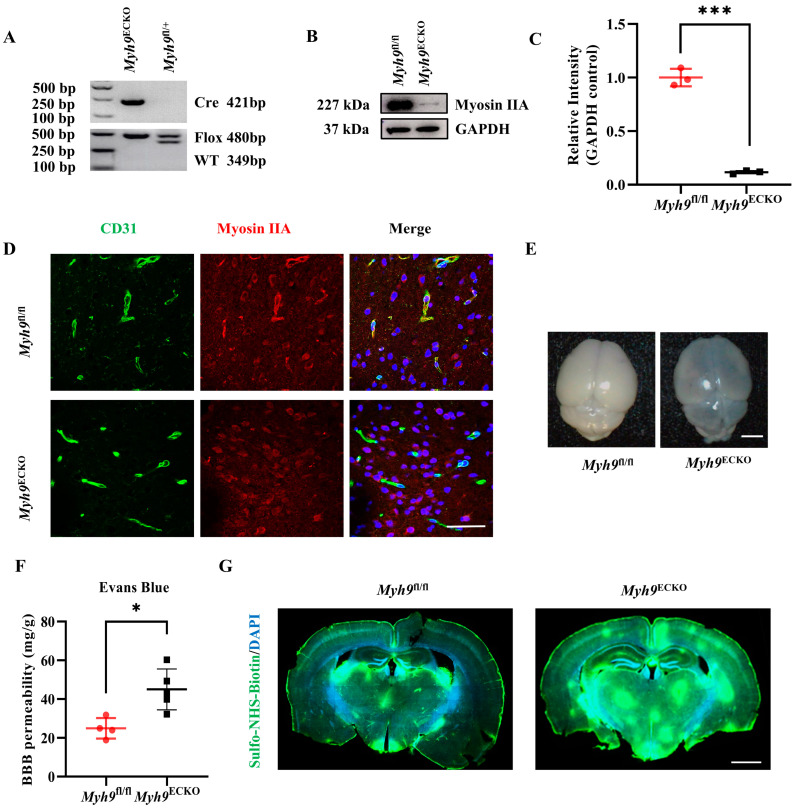
Brain endothelial deletion of Myosin IIA impairs the integrity of the BBB in mice. (**A**) Representative PCR gel image for genotyping mice using tail genomic DNA, showing floxed allele band at 480 bp, WT allele band at 349 bp, and Cre-positive band at 421 bp. (**B**) Representative Western blot image, showing Myosin IIA protein levels in primary brain ECs from *Myh9*^fl/fl^ and *Myh9*^ECKO^ mice (*n* = 3). (**C**) Statistical analysis of Myosin IIA protein levels in primary brain ECs from *Myh9*^fl/fl^ and *Myh9*^ECKO^ mice (*n* = 3). *** *p* < 0.001; Student’s *t*-test. (**D**) Representative immunofluorescence staining of brain tissue sections from *Myh9*^fl/fl^ and *Myh9*^ECKO^ mice, showing CD31 (green), Myosin IIA (red), and DAPI (blue). Scale bar: 50 μm. (**E**) Representative image of brain tissues from *Myh9*^fl/fl^ and *Myh9*^ECKO^ mice following intravenous injection of Evans blue. Scale bar: 5 mm. (**F**) Statistical analysis of total Evans blue tracer leakage into the brain parenchyma of *Myh9*^fl/fl^ and *Myh9*^ECKO^ mice (*Myh9*^fl/fl^, *n* = 4; *Myh9*^ECKO^, *n* = 5). * *p* < 0.05; Student’s *t*-test. (**G**) Representative immunofluorescence staining of brain sections from *Myh9*^fl/fl^ and *Myh9*^ECKO^ mice following intravenous injection of Sulfo-NHS-Biotin, showing Sulfo-NHS-Biotin (green) and DAPI (blue). Scale bar: 1 mm.

To investigate the role of Myosin IIA in the brain endothelium, we aimed to create genetically modified mice with targeted disruption of Myosin IIA in cerebral ECs. SP-A-Cre mice is a strain that has been previously shown to specifically express Cre recombinase activity in the ECs of the mouse brain vasculature during both embryonic and adult stages [40,41]. This strain has been recognized as a useful genetic tool to study the function of the BBB and its associated mechanisms [40,41,42,43,44]. Subsequently, we crossed these mice with *Myh9*^fl/fl^ mice to produce brain endothelium-specific *Myh9* knockout mice (*SP-A*-Cre^+/−^ *Myh9*^fl/fl^, *Myh9*^ECKO^), as depicted in Figure 1A and Figure S1A.

To verify the knockout efficiency of *Myh9* in the cerebral ECs of *Myh9*^ECKO^ mice, we initially isolated primary cerebral ECs from both *Myh9*^fl/fl^ and *Myh9*^ECKO^ mice using flow cytometry. As shown in Figure 1, Myosin IIA expression is significantly decreased in the *Myh9*^ECKO^ mice compared to the controls, with a knockout efficiency approaching 90% (Figure 1B,C). Further, RNA was extracted and subjected to RT-PCR, revealing a decrease in *Myh9* mRNA levels in the cerebral endothelium of *Myh9*^ECKO^ mice relative to the controls (Figure S1B). Immunofluorescence analysis on brain sections from both mouse genotypes indicated that Myosin IIA expression was reduced specifically in cerebral ECs of *Myh9*^ECKO^ mice, with no impact on its expression in other brain cells (Figure 1D). Additionally, to further validate the specificity of our knockout model, we isolated liver sinusoidal endothelial cells (LSECs) and lung endothelial cells (LECs) from *Myh9*^fl/fl^ and *Myh9*^ECKO^ mice and performed the Western blot analysis. The results demonstrate no significant changes in Myosin IIA expression in these peripheral tissues, confirming that the knockout specifically affects brain endothelial cells (Figure S1C).

The genotypic distribution of the progeny adhered to Mendelian ratios, with no adverse effects on the survival or reproductive capabilities of the *Myh9*^ECKO^ mice (Figure S1C). To determine if the endothelium-specific knockout of *Myh9* influences cerebral vascular morphology, brain tissues from 8-week-old *Myh9*^fl/fl^ and *Myh9*^ECKO^ mice were sectioned and stained for CD31 to visualize blood vessels (Figure S2A). The statistical analyses revealed no significant differences in vessel density, branching, or length in the hippocampus, cortex, or striatum between the *Myh9*^ECKO^ mice and controls (Figure S2B–D). These findings indicate that a deficiency in endothelial Myosin IIA does not result in noticeable alterations in gross cerebral vascular morphology.

*Myh9*^ECKO^ mice were administered 2.5% Evans blue intravenously, and after 18 h of circulation, an increased leakage of the dye into the brain parenchyma was detected in these mice compared to the controls (Figure 1E,F). To further assess the changes in BBB permeability, additional tracer molecules were also used. An increased accumulation of Sulfo-NHS-Biotin (550 Da) in the brain parenchyma outside the vessels was also observed in these mice compared to the controls (Figure 1G). These results demonstrate that Myosin IIA deficiency in cerebral endothelium compromises the integrity of the BBB. Furthermore, inducing Myosin IIA deficiency in adult mice after tamoxifen administration resulted in similar BBB disruptions, evidenced by increased Evans blue leakage in the brain parenchyma of *Myh9*^iECKO^ (*Cdh5*-CreER^+/−^ *Myh9*^fl/fl^) mice (Figure S3), confirming the critical role of Myosin IIA in maintaining BBB integrity.

### 3.2. Cerebral Endothelial Deletion of Myosin IIA Increases Seizure Susceptibility and Seizure-Induced Mortality

The disruption of the BBB not only serves as a marker for brain disease pathology but also accelerates the progression of neurological disorders. Previous studies have demonstrated that endothelial abnormalities or BBB deficiencies can provoke epileptic seizures and exacerbate the resultant damage [45,46]. Consequently, we hypothesized that *Myh9*^ECKO^ mice, with compromised BBB integrity, will exhibit more severe damage in an epilepsy model.

**Figure 2:**
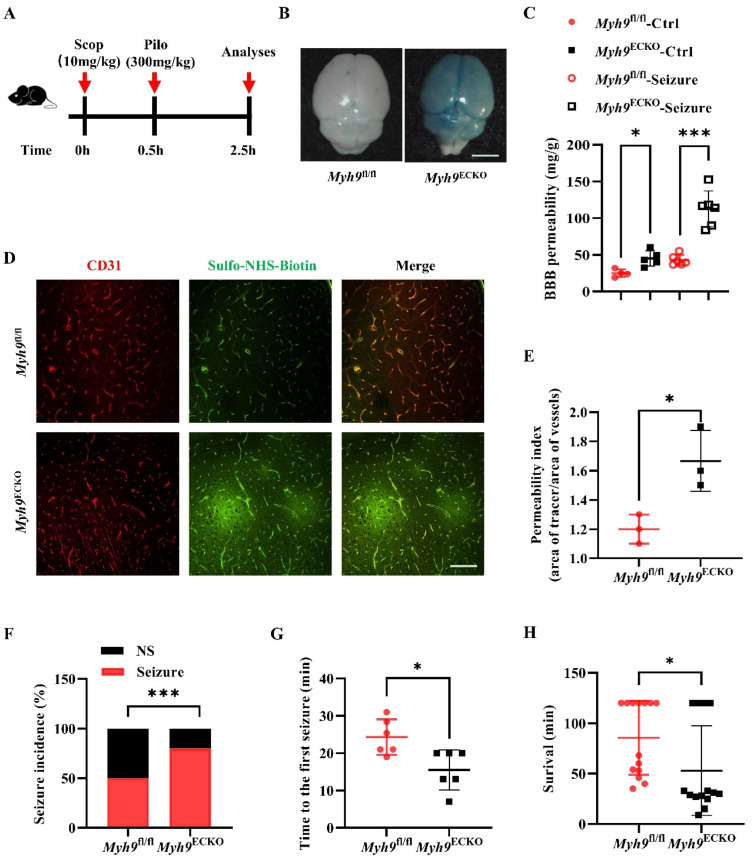
Brain endothelial deletion of Myosin IIA increases seizure susceptibility and seizure-induced mortality. (**A**) Experimental design for a mouse epilepsy model, involving intraperitoneal injection of scopolamine followed by pilocarpine 30 min later, with mouse behavior monitored over the next 120 min. (**B**) Representative images of Evans blue tracer leakage in brain tissues of *Myh9*^fl/fl^ and *Myh9*^ECKO^ mice after pilocarpine-induced epilepsy. Scale bar: 5 mm. (**C**) Statistical analysis of total Evans blue tracer leakage into the brain tissues of *Myh9*^fl/fl^ and *Myh9*^ECKO^ mice post-pilocarpine administration (*n* = 6). * *p* < 0.05, *** *p* < 0.001; one-way ANOVA test. (**D**) Representative immunofluorescence co-staining images of brain sections from *Myh9*^fl/fl^ and *Myh9*^ECKO^ mice post-pilocarpine administration, showing CD31 (red) and Sulfo-NHS-Biotin (green). Scale bar: 100 μm. (**E**) Statistical analysis of Sulfo-NHS-Biotin leakage index in brain tissues of *Myh9*^fl/fl^ and *Myh9*^ECKO^ mice after pilocarpine-induced epilepsy (*n* = 3). * *p* < 0.05; Student’s *t*-test. (**F**) Statistical analysis of the incidence of seizure occurrence in *Myh9*^fl/fl^ and *Myh9*^ECKO^ mice after pilocarpine administration (NS, no seizure group; Seizure, seizure group; *n* = 14). *** *p* <0.001; Fisher’s exact test. (**G**) Time to first seizure onset in *Myh9*^fl/fl^ and *Myh9*^ECKO^ mice following pilocarpine administration (*n* = 6). * *p* < 0.05; Student’s *t*-test. (**H**) Survival time of *Myh9*^fl/fl^ and *Myh9*^ECKO^ mice after pilocarpine administration, with a total monitoring duration of 120 min (*n* = 14). * *p* < 0.05; Student’s *t*-test.

To investigate this hypothesis, we initially established an epilepsy model using Pilocarpine (Pilo), a classical method for inducing seizures. Mice were first administered Evans blue or Sulfo-NHS-Biotin tracers intravenously, followed by intraperitoneal injection of scopolamine, and then Pilo to induce seizures. Over the 120 min following Pilo administration, the mice were closely monitored. Seizure scores were recorded, along with the timing of seizures and mortality (Figure 2A). The results indicate that, compared to the controls, *Myh9*^ECKO^ mice showed increased brain tissue levels of Evans blue and a higher proportion of Sulfo-NHS-Biotin leakage from cerebral vessels, suggesting enhanced BBB permeability in this Pilo-induced epilepsy model (Figure 2B–E). A further analysis revealed that *Myh9*^ECKO^ mice had a higher rate of seizures (Figure 2F) and experienced their first seizure in a shorter period (Figure 2G). Additionally, a higher mortality rate following seizures was observed in *Myh9*^ECKO^ mice compared to the controls, with most succumbing within 120 min post-administration (Figure 2H). These findings suggest that *Myh9*^^ECKO^ mice are more susceptible to Pilo-induced epilepsy and suffer more severe outcomes.

In summary, the endothelial-specific knockout of *Myh9* results in the disruption of BBB integrity, which in turn exacerbates damage caused by BBB-associated diseases, such as epilepsy.

### 3.3. Cerebral Endothelial Deletion of Myosin IIA Disrupts Brain Endothelial Tight Junctions

The findings presented above demonstrate that the endothelial-specific knockout of *Myh9* disrupts the integrity of the BBB, and this disruption exacerbates damage caused by brain diseases. However, the specific mechanisms by which Myosin IIA regulates BBB integrity remain unclear and warrant further investigation. ECs in the brain are connected by specialized tight junctions and express crucial transport proteins, playing a vital role in maintaining the BBB. Additionally, extensive research has shown that the normal function of the BBB is maintained by a complex interplay among Ecs, pericytes, astrocytes, and the basement membrane, with Ecs regulating the surrounding microvascular cells to support the BBB [10,11,12,13,14]. We primarily explore the specific mechanisms by which Myosin IIA deficiency in the brain endothelium contributes to BBB damage from two aspects: first, whether Myosin IIA deficiency in brain endothelium disrupts tight junction structures or alters the expression of critical endothelial molecules; second, whether Myosin IIA deficiency affects the function of pericytes and astrocytes within the BBB.

**Figure 3:**
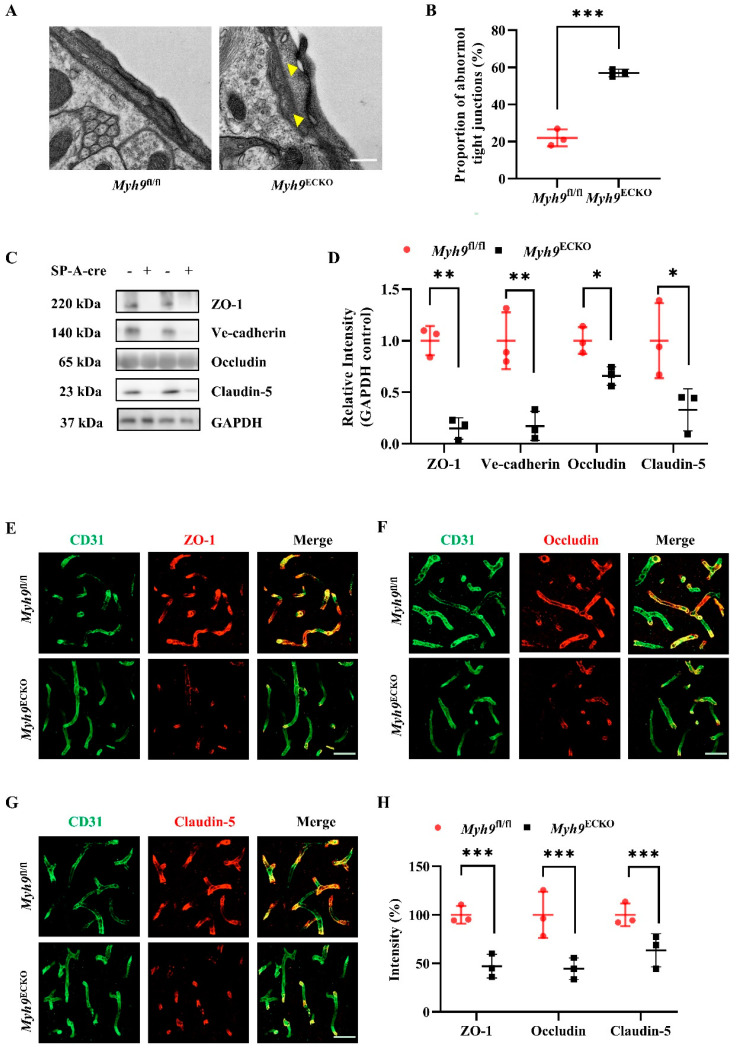
Deletion of brain endothelial Myosin IIA downregulates junctional proteins of the BBB. (**A**) Representative electron microscopy image of vascular endothelial tight junction structures in brain sections from *Myh9*^fl/fl^ and *Myh9*^ECKO^ mice, with yellow arrows indicating disrupted tight junctions. Scale bar: 200 nm. (**B**) Statistical analysis of the proportion of abnormal tight junction structures in brain sections from *Myh9*^fl/fl^ and *Myh9*^ECKO^ mice (*n* = 3). *** *p* < 0.001; Student’s *t*-test. (**C**) Western blot analysis of ZO-1, Ve-cadherin, Occludin, and Claudin-5 protein levels in primary brain ECs from *Myh9*^fl/fl^ and *Myh9*^ECKO^ mice. (**D**) Statistical analysis of protein expression levels of ZO-1, Ve-cadherin, Occludin, and Claudin-5 in primary brain ECs from *Myh9*^fl/fl^ and *Myh9*^ECKO^ mice (*n* = 3). * *p* < 0.05, ** *p* < 0.01; Student’s *t*-test. (**E**) Representative immunofluorescence co-staining image of brain sections from *Myh9*^fl/fl^ and *Myh9*^ECKO^ mice, showing CD31 (green) and ZO-1 (red). Scale bar: 50 μm. (**F**) Representative immunofluorescence co-staining image of brain sections from *Myh9*^fl/fl^ and *Myh9*^ECKO^ mice, showing CD31 (green) and Occludin (red). Scale bar: 50 μm. (**G**) Representative immunofluorescence co-staining image of brain sections from *Myh9*^fl/fl^ and *Myh9*^ECKO^ mice, showing CD31 (green) and Claudin-5 (red). Scale bar: 50 μm. (**H**) Statistical analysis of colocalization of ZO-1, Occludin, Claudin-5, and CD31 in brain sections from *Myh9*^fl/fl^ and *Myh9*^ECKO^ mice (*n* = 3). *** *p* < 0.001; Student’s *t*-test.

Extensive research has demonstrated that specialized cellular connections between cerebral vascular Ecs play a crucial role in regulating the integrity of the BBB [20,23,47,48]. Therefore, we hypothesized that the cellular connections among the Ecs of *Myh9*^ECKO^ mice might be compromised. Using electron microscopy to observe the ultrastructure of cerebral vessels, we found noticeable discontinuities in the tight junction structures between adjacent Ecs in *Myh9*^ECKO^ mice compared to the controls (Figure 3A,B). The structure and function of endothelial tight junctions are associated with the expression and proper localization of various junction-related molecules, such as ZO-1, Occludin, and Claudin-5. The abnormal junction structures observed in the ECs of *Myh9*^ECKO^ mice under electron microscopy suggest potential alterations in the expression of these constituent molecules. We isolated brain ECs from 8-week-old *Myh9*^fl/fl^ and *Myh9*^ECKO^ mice using flow cytometry, followed by the extraction of proteins and mRNA from these cells to perform Western blot and RT-PCR analyses. The expression levels of tight junction molecules, such as ZO-1, Occludin, and Claudin-5, were investigated. The results indicate a decrease in both protein and mRNA levels of these cellular junction components (Figure 3C,D and Figure S4). Additionally, the co-immunofluorescence staining results of ZO-1, Claudin-5, Occludin, and CD31 showed a significant reduction in the coverage of these junction proteins on the brain endothelium (Figure 3E–H). These findings suggest that the increased permeability of the BBB in *Myh9*^ECKO^ mice is associated with the disruption of endothelial cell junction structures, caused by the reduction in mRNA and protein levels of various junction constituents.

The brain endothelium expresses a variety of crucial transport proteins that mediate the translocation of essential substances, such as amino acids and glucose, thus playing a key role in regulating the cerebral microenvironment. Among these, the endothelial expression of Glut1 facilitates glucose transport, providing energy to the brain; deficiencies in Glut1 are linked to brain development disorders and BBB damage [49,50]. Accordingly, we conducted immunofluorescence staining for Glut1 on mouse brain sections, which revealed no difference in Glut1 protein expression between the blood vessels of *Myh9*^ECKO^ mice and the controls (Figure S6A,B).

We further investigated whether Myosin IIA deficiency in the brain endothelium disrupts the normal structure of the basement membrane, pericytes, and astrocytes within the BBB. The immunofluorescence analysis revealed that the morphology and coverage of basement membranes, marked by Laminin on blood vessels, in *Myh9*^ECKO^ mice were similar to those in the control group (Figure S6A,B). Additionally, there were no differences in the morphology and coverage of pericytes, marked by Pdgfrβ around blood vessels, between *Myh9*^ECKO^ mice and the controls (Figure S6C,D). Furthermore, no astrocyte proliferation, marked by glial fibrillary acidic protein (GFAP), was observed in the brain parenchyma of *Myh9*^ECKO^ mice (Figure S6E). Brain tissues from 8-week-old *Myh9*^fl/fl^ and *Myh9*^ECKO^ mice were also prepared for electron microscopy to observe the ultrastructure of mouse cerebral vessels. Similar to the controls, the morphology of pericytes around the blood vessels of *Myh9*^ECKO^ mice was normal, adhering to the vessel walls; the morphology of astrocytes was normal, and the basement membrane structures between them and ECs were intact (Figure S6F) [51]. These findings indicate that the structures of the basement membrane, pericytes, and astrocytes in *Myh9*^ECKO^ mice are normal.

### 3.4. Myosin IIA Mediates the Transcription of the Ctnnb1 Gene and Interacts with Its Protein β-Catenin

**Figure 4:**
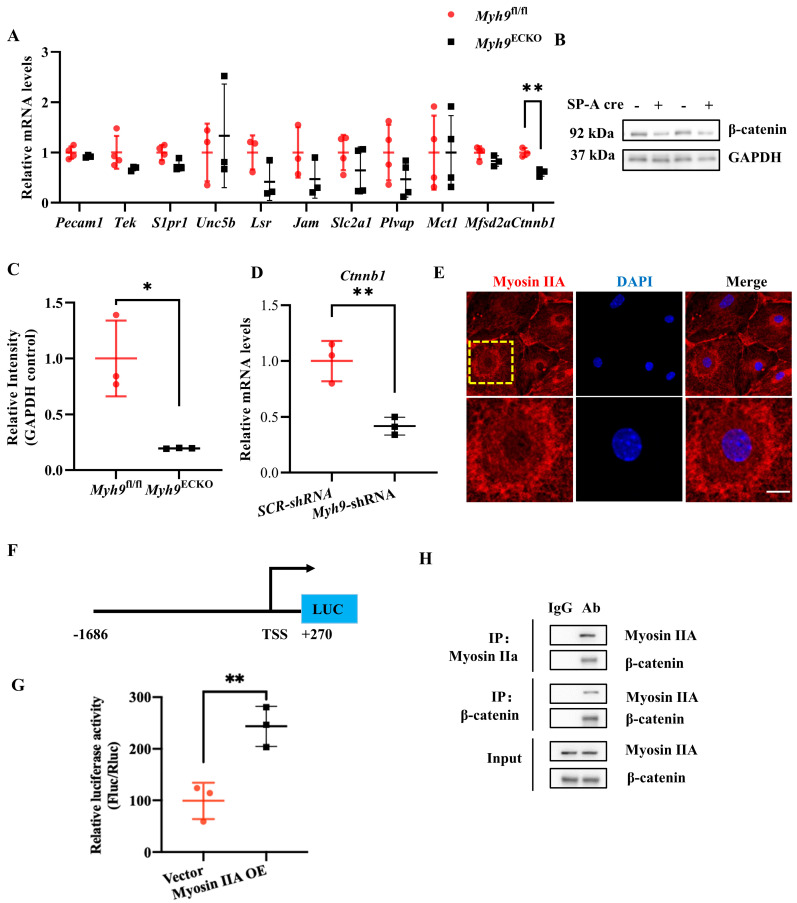
Myosin IIA mediates the transcription of *Ctnnb1* gene and interacts with its protein β-catenin. (**A**) RNA was extracted from primary brain ECs of *Myh9*^fl/fl^ and *Myh9*^ECKO^ mice, and RT-PCR was conducted to assess mRNA expression levels of key molecules regulating the blood–brain barrier (*n* = 3–4). ** *p* < 0.01; Student’s *t*-test. (**B**) Western blot analysis of β-catenin protein levels in primary brain ECs from *Myh9*^fl/fl^ and *Myh9*^ECKO^ mice. (**C**) Statistical analysis of β-catenin protein levels in primary brain ECs from *Myh9*^fl/fl^ and *Myh9*^ECKO^ mice (*n* = 3). * *p* < 0.05; Student’s *t*-test. (**D**) RT-PCR analysis of *Ctnnb1* mRNA levels in SCR-shRNA and *Myh9*-shRNA group cells (*n* = 3). ** *p* < 0.01; Student’s *t*-test. (**E**) Representative immunofluorescence staining of Myosin IIA in HCMEC/D3 brain ECs, with Myosin IIA (red) and DAPI (blue). Scale bar: 20 μm. (**F**) Construction strategy for a luciferase reporter plasmid containing the Ctnnb1 gene promoter. (**G**) Brain ECs overexpressing either control or Myosin IIA were co-transfected with the *Ctnnb1* gene promoter luciferase reporter plasmid and Renilla luciferase reporter plasmid. Cells were collected 24 h post-transfection to measure their activity (*n* = 3). ** *p* < 0.01; Student’s *t*-test. (**H**) Interaction between Myosin IIA and β-catenin. Once bEnd.3 brain ECs reached a confluent monolayer, cells were lysed, proteins were harvested, and co-immunoprecipitation was performed to detect interactions between Myosin IIA and β-catenin proteins.

To delve deeper into these mechanisms, this research, drawing on the previous literature, identified key genes involved in regulating BBB functions related to cerebral endothelium. These genes were considered candidate downstream molecules affected by Myosin IIA [52,53,54]. Subsequent experiments involved isolating primary ECs from 8-week-old *Myh9*^fl/fl^ and *Myh9*^ECKO^ mice using flow cytometry, extracting RNA from these cells, and conducting RT-PCR to systematically screen the transcription levels of the aforementioned candidates. The results show that most genes did not differ between *Myh9*^fl/fl^ and *Myh9*^ECKO^ mice, except for *Ctnnb1* (encoding β-catenin), which was significantly downregulated (Figure 4A).

In light of these findings, further experiments were conducted using brain ECs isolated via flow cytometry to perform Western blot analyses. Consistent with the RT-PCR results, β-catenin protein expression was found to be downregulated in the brain ECs of *Myh9*^ECKO^ mice compared to the controls (Figure 4B,C). Previous research has reported that mice with endothelial-specific β-catenin deficiencies induced by tamoxifen exhibited spontaneous epileptic symptoms, along with neuronal damage, multifocal intracerebral hemorrhages, and central nervous system inflammation, leading to death within 16 days post-induction. These studies suggest that the β-catenin signaling pathway in ECs is crucial for maintaining BBB integrity and central nervous system homeostasis in adult mice [52,54]. These results imply that β-catenin may be a key effector molecule through which Myosin IIA regulates BBB integrity.

Building on previous findings, we explored multiple potential mechanisms by which Myosin IIA could regulate β-catenin. Initially, we examined whether Myosin IIA affects the mRNA levels of *Ctnnb1* in the brain endothelial cell line bEnd.3. Cells were infected with *Myh9*-shRNA virus to interfere with Myosin IIA expression and, after six days, cells from the SCR-shRNA control group and the *Myh9*-shRNA group were collected for RT-PCR to measure mRNA expression levels. The results show that, consistent with in vivo findings, *Ctnnb1* mRNA levels decreased in bEnd.3 cells following Myosin IIA knockdown (Figure 4D).

Previous research has demonstrated that, in a gastric cancer model in mice, Myosin IIA can bind to the promoter region of the *Ctnnb1* gene in gastric cancer cells, mediating its transcriptional expression [55]. To ascertain whether Myosin IIA also enters the nucleus and participates in transcriptional regulation signaling in brain ECs, we employed immunofluorescence staining to examine the expression of Myosin IIA in the nuclei of these cells. The results indicate that Myosin IIA is expressed in both the nucleus and cytoplasm of brain ECs (Figure 4E). Furthermore, to investigate whether Myosin IIA can regulate transcription by binding to the promoter region of *Ctnnb1* in brain ECs, a luciferase reporter plasmid containing the *Ctnnb1* promoter region was constructed, following previous reports (Figure 4F). This plasmid, along with a Renilla luciferase reporter plasmid and a plasmid overexpressing Myosin IIA, was co-transfected into brain ECs. After 24 h, cells were collected, and the luminescence signal was measured. The dual-luciferase reporter assay results show that, compared to the control group, the transcriptional activity of *Ctnnb1* was higher in the brain ECs overexpressing Myosin IIA (Figure 4G). These findings demonstrate that Myosin IIA can mediate the transcription of the *Ctnnb1* gene in brain ECs.

However, it does not rule out the possibility that Myosin IIA may influence the protein expression levels of β-catenin through other mechanisms. Extensive research has shown that Myosin IIA, as a cytoskeletal protein, can bind to various intracellular molecules and perform multiple biological functions. Previous proteomic analyses have demonstrated that Myosin IIA can bind to β-catenin in ECs, an interaction that is highly relevant to our hypothesis concerning endothelial junction integrity. Building on these findings, we further explored this interaction in brain endothelial cells. Immunoprecipitation experiments using bEnd.3 cells confirmed a direct interaction between endogenous Myosin IIA and β-catenin (Figure 4H), supporting the hypothesis that Myosin IIA modulates β-catenin function, which is critical for maintaining tight junctions and endothelial barrier integrity.

These results suggest that Myosin IIA mediates the transcription of *Ctnnb1* and binds to its protein β-catenin to regulate the expression and function of β-catenin.

### 3.5. Overexpression of β-Catenin Ameliorates BBB Leakage in Myh9^ECKO^ Mice

**Figure 5:**
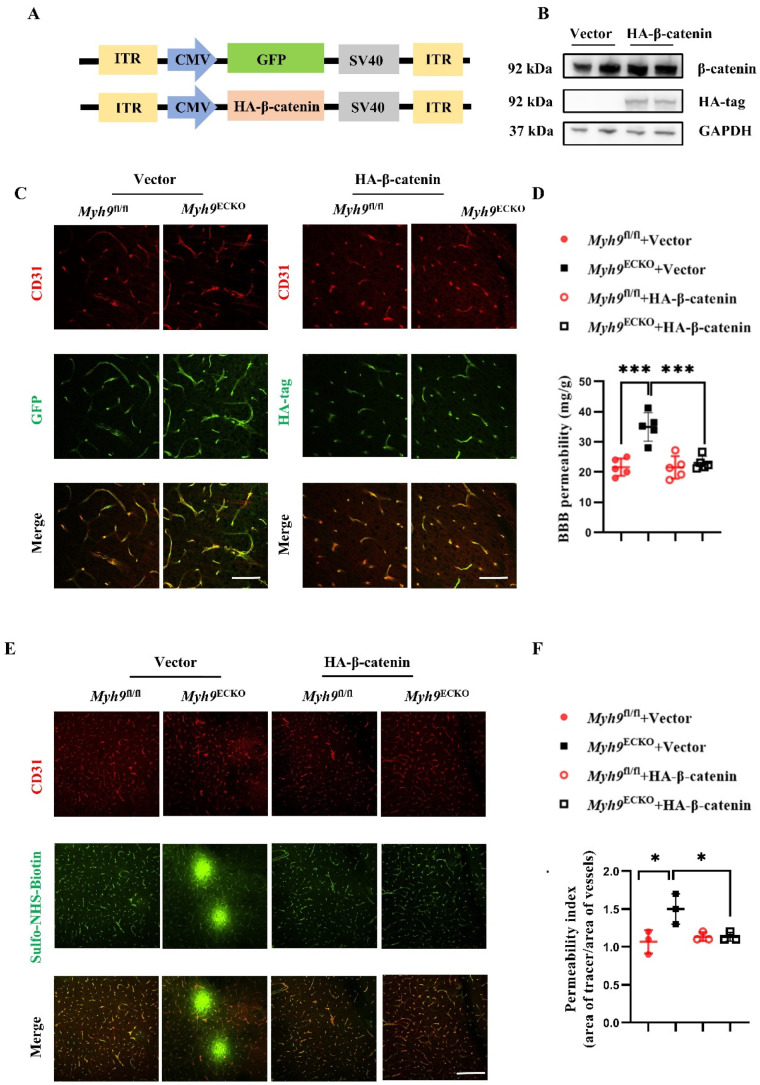
Overexpression of β-catenin ameliorates BBB leakage in *Myh9*^ECKO^ mice. (**A**) Construction strategy for AAV-GFP and AAV-HA-β-catenin overexpression plasmids. (**B**) Transfection of AAV-GFP and AAV-HA-β-catenin plasmids into 293T cells; 48 h later, cell proteins were harvested and analyzed by Western blot to assess β-catenin protein levels. (**C**) Representative immunofluorescence co-staining images of brain sections from *Myh9*^fl/fl^ and *Myh9*^ECKO^ mice 30 days post-injection with AAV-GFP virus showing CD31 (red) and GFP (green); and 30 days post-injection with AAV-HA-β-catenin virus showing CD31 (red) and HA-tag (green). Scale bar: 100 μm. (**D**) Intravenous injection of AAV-GFP or AAV-HA-β-catenin virus in *Myh9*^fl/fl^ and *Myh9*^ECKO^ mice; 30 days later, Evans blue was injected intravenously, and the total amount of Evans blue in the brain tissues of different treatment groups was quantitatively analyzed *(n* = 5). *** *p* < 0.001; one-way ANOVA test. (**E**) Intravenous injection of AAV-GFP or AAV-HA-β-catenin virus in *Myh9*^fl/fl^ and *Myh9*^ECKO^ mice; 30 days later, Sulfo-NHS-Biotin was injected, and brain sections were co-stained for CD31 (red) and Sulfo-NHS-Biotin (green). Scale bar: 100 μm. (**F**) Statistical analysis of Sulfo-NHS-Biotin leakage index in brain tissues of *Myh9*^fl/fl^ and *Myh9*^ECKO^ mice 30 days post-injection with AAV-GFP or AAV-HA-β-catenin virus (*n* = 3). Scale bar: 200 μm. * *p* < 0.05; one-way ANOVA test.

In this section, we further investigated whether the overexpression of β-catenin in vivo could reduce the permeability of the BBB in *Myh9*^ECKO^ mice. The specific intervention in the brain endothelium remains a significant scientific challenge in the field. Previous studies have reported on AAV-BI30, a new serotype of AAV that can specifically and efficiently transduce ECs throughout the central nervous system, achieving stable expression in vitro human microvascular ECs, as well as in mouse and rat models [56,57]. To achieve the overexpression of β-catenin in mouse brain ECs using the AAV-BI30 system, we constructed a β-catenin overexpression plasmid tagged with HA (Figure 5A). The plasmid was transfected into 293T cells, and proteins were harvested 48 h later for Western blot analysis, which confirmed the successful expression of β-catenin in 293T cells (Figure 5B).

Utilizing the AAV-BI30 system, we packaged and purified an AAV-HA-β-catenin virus for overexpressing β-catenin in the brain endothelium of mice, along with an AAV-GFP virus as a control. Thirty-day-old *Myh9*^fl/fl^ and *Myh9*^ECKO^ mice were intravenously injected with these AAV viruses. After 30 days, the mice were anesthetized and euthanized, and brain tissues were collected for sectioning. The immunofluorescence analysis of the brain tissues revealed that in both *Myh9*^fl/fl^ and *Myh9*^ECKO^ mice, a GFP fluorescence signal was detectable in most brain vessels following AAV-GFP virus injection. HA-β-catenin overexpression signals were detected in most brain vessels of both *Myh9*^fl/fl^ and *Myh9*^ECKO^ mice after injection with the AAV-HA-β-catenin virus (Figure 5C). Additionally, the Western blot results of brain ECs show that the protein expression of key tight junction proteins, including Claudin-5, Occludin, and ZO-1, was increased in *Myh9*^ECKO^ mice after AAV-HA-β-catenin treatment (Figure S7A).

Subsequently, we investigated whether overexpressing β-catenin in the brain endothelium of mice could decrease the permeability of the BBB in *Myh9*^ECKO^ mice. Four-week-old *Myh9*^fl/fl^ and *Myh9*^ECKO^ mice were intravenously injected with the AAV virus. After 30 days, Evans blue and Sulfo-NHS-Biotin tracers were administered intravenously to analyze the leakage of these compounds in the mice’s brain tissue. The results demonstrate that, compared to the GFP overexpression control group, the total amount of Evans blue that leaked into the brain parenchyma was significant, and the leakage of Sulfo-NHS-Biotin out of the brain vessels was reduced (Figure 5D–F). These findings suggest that the overexpression of β-catenin in the brain endothelium of *Myh9*^ECKO^ mice can improve the function of the BBB.

### 3.6. Overexpression of β-Catenin Ameliorates Epileptic Damage in Myh9^ECKO^ Mice

**Figure 6:**
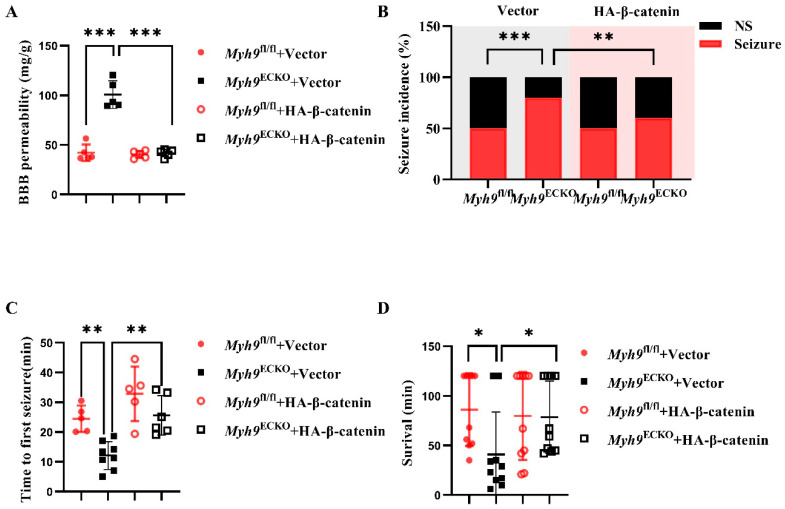
Overexpression of β-catenin in cerebral endothelium of *Myh9*^ECKO^ mice ameliorates epilepsy-induced injury. (**A**) Statistical analysis of the total amount of Evans blue tracer in the brain tissues of *Myh9*^fl/fl^ and *Myh9*^ECKO^ mice injected intravenously with AAV-GFP or AAV-HA-β-catenin viruses, followed by pilocarpine-induced epilepsy 30 days later (*n* = 5). *** *p* < 0.001; one-way ANOVA test. (**B**) Statistical analysis of the incidence of epilepsy in *Myh9*^fl/fl^ and *Myh9*^ECKO^ mice after intravenous injection of AAV-GFP or AAV-HA-β-catenin viruses and subsequent pilocarpine administration 30 days later (NS, no seizure group; Seizure, seizure group; *n* = 10). ** *p* < 0.01, *** *p* < 0.001; Fisher’s exact test. (**C**) Statistical analysis of the time to first seizure in *Myh9*^fl/fl^ and *Myh9*^ECKO^ mice treated with AAV-GFP or AAV-HA-β-catenin viruses and subsequently induced with pilocarpine (*Myh9*^fl/fl^+Vector and *Myh9*^fl/fl^+HA-β-catenin, *n* = 5; *Myh9*^ECKO^, *n* = 8; *Myh9*^ECKO^ +HA-β-catenin, *n* = 6). ** *p* < 0.01; one-way ANOVA test. (**D**) Statistical analysis of survival time for *Myh9*^fl/fl^ and *Myh9*^ECKO^ mice injected with AAV-GFP or AAV-HA-β-catenin viruses and then induced with pilocarpine 30 days later, with a total monitoring duration of 120 min (*n* = 10). * *p* < 0.05; one-way ANOVA test.

In this section, we hypothesized that overexpressing β-catenin in the brain endothelial of *Myh9*^ECKO^ mice could ameliorate the damage caused by epileptic seizures. To test this hypothesis, an epilepsy model was induced by drug administration in mice 30 days after AAV injection. The results show that, compared to the GFP overexpression control group, *Myh9*^ECKO^ mice overexpressing β-catenin in their brain endothelium exhibited a significant reduction in the total amount of Evans blue that leaked into the brain parenchyma, a lower rate of epileptic seizures, increased time to the first seizure, and a reduced mortality rate from epilepsy (Figure 6). These findings suggest that the overexpression of β-catenin in the brain endothelium of *Myh9*^ECKO^ mice can decrease their susceptibility to epilepsy and mitigate the damage caused by epileptic seizures.

## 4. Discussion

The BBB not only protects against harmful substances and inflammatory cells entering the brain parenchyma from the bloodstream but also facilitates the transport of nutrients essential for normal physiological activities of brain tissues. The integrity of the BBB is critical for maintaining the homeostasis of the brain microenvironment, with brain ECs playing a significant role in regulating the structure and function of the BBB. In mouse brain tissue, Myosin IIB is primarily expressed in neurons, Myosin IIC is expressed at relatively low levels across various cell types, and Myosin IIA is highly expressed in the brain endothelium, indicating a specialized role in these cells [58]. The BBB integrity of *Myh9*^ECKO^ mice was assessed using a variety of tracers, such as Evans blue and biotin; we observed significant tracer leakage in the brain tissue of *Myh9*^ECKO^ mice compared to the controls, indicating that endothelial Myosin IIA deficiency compromises BBB integrity. Interestingly, studies have shown that during ischemia-reperfusion-induced injury, the deficiency of endothelial Myosin IIA may ameliorate brain damage caused by oxidative stress [31]. Increased Myosin IIA expression and activity under oxidative stress leads to endothelial contraction, impairing barrier function. For instance, Gong et al. demonstrated that the conditional knockdown of Myosin IIA in endothelial cells during ischemia-reperfusion injury improved BBB integrity and reduced brain damage. These findings suggest that, while Myosin IIA is essential for maintaining vascular stability under physiological conditions, its upregulation and excessive activity in response to pathological stressors, such as ischemia or oxidative stress, can contribute to BBB dysfunction. This contrasting role of Myosin IIA under physiological and pathological conditions underscores its context-dependent nature. Under normal physiological conditions, Myosin IIA stabilizes endothelial cell junctions and regulates cell contractility to maintain BBB integrity. However, under pathological conditions like ischemia-reperfusion injury, its overactivation can lead to increased endothelial cell contraction, BBB breakdown, and exacerbation of disease. Understanding how Myosin IIA’s function is modulated in these different contexts is critical for developing therapeutic strategies that can either enhance its protective role or inhibit its harmful effects under stress. Our findings reveal that, under physiological conditions, a reduction in Myosin IIA expression and activity also leads to increased basal BBB permeability, emphasizing the importance of Myosin IIA in maintaining BBB homeostasis through its regulatory mechanisms under both physiological and pathological conditions.

In this study, we demonstrate that endothelial Myosin IIA is crucial for the maintenance of BBB integrity under physiological conditions, and its deficiency exacerbates the pathogenesis of epilepsy through β-catenin. Considering the importance of BBB integrity, it is relevant to discuss the potential impact of a pro-oxidant environment, such as that found in inflammation and oxidative stress [59]. Oxidative stress has been shown to alter the molecular profile of myosins, potentially affecting their structure and function [60,61]. These modifications could influence the activity of β-catenin, which is a key mechanism identified in our study. Additionally, oxidative stress is implicated in age-related diseases that involve BBB impairment [62,63], suggesting that changes in endothelial Myosin IIA in response to a pro-oxidant environment may exacerbate conditions such as epilepsy. However, the precise mechanisms by which oxidative stress affects Myosin IIA and its interaction with β-catenin, as well as their impact on BBB integrity, require further investigation. These insights underline the significance of endothelial Myosin IIA in BBB maintenance and suggest that targeting Myosin IIA and its associated pathways may offer novel therapeutic strategies, although more research is needed to fully understand the underlying processes

Mutations in the *MYH9* gene cause *MYH9*-related diseases (*MYH9*-RD), resulting in the production of a dysfunctional Myosin IIA protein rather than a complete loss of function [64]. *MYH9*-RD is an autosomal dominant genetic disease characterized by thrombocytopenia and large platelets. Initial research on *MYH9*-RD primarily focused on hematological abnormalities, such as coagulation dysfunctions, with limited studies on neurological disorders. However, subsequent studies have reported that some *MYH9*-RD patients develop cerebral conditions, including subarachnoid hemorrhage [65]. Correlation analysis in clinical settings also suggests that *MYH9* is a susceptibility gene linked to autism, schizophrenia, and intellectual disabilities [66], indicating that *MYH9*-RD patients may benefit from preventive monitoring for neurological diseases. In our study, we provide insights into the importance of Myosin IIA in maintaining BBB integrity, suggesting that alterations in Myosin IIA, whether from genetic mutations or other mechanisms, may contribute to BBB dysfunction. While we did not specifically investigate *MYH9*-RD, these findings underscore the need for further research into how *MYH9* mutations may impact BBB integrity and contribute to central nervous system pathologies. Given that specific mutations in *MYH9* influence its biological functions, it remains to be explored whether common mutations found in *MYH9*-RD patients also contribute to BBB damage. Further research, particularly utilizing more human data, is necessary to better understand and potentially diagnose and treat neurological conditions in *MYH9*-RD patients. Moreover, although the expression and function of Myosin IIA are largely conserved between humans and mice [25], species-specific differences in regulation and localization may exist. Therefore, while our mouse model provides valuable insights, further studies are required to determine whether endothelial Myosin IIA plays a similar role in the human BBB. Understanding these potential differences is essential for translating our findings into therapeutic applications for human neurological conditions.

Brain ECs, as the structural foundation of the BBB, are crucial for maintaining its integrity. Investigating the specific mechanisms through which brain ECs preserve BBB integrity has been a focal point in the field [67,68,69,70,71]. We identify Myosin IIA as a novel molecule maintaining BBB integrity, offering new insights into research related to the homeostasis of the brain microenvironment. We analyzed the ultrastructure of cerebral blood vessels in the BBB of *Myh9*^ECKO^ mice using electron microscopy. It was found that the tight junction structures of brain ECs in *Myh9*^ECKO^ mice were significantly disrupted, although the structures of pericytes, astrocytes, and the basement membrane remained normal. Additionally, there was a reduction in the mRNA and protein levels of cellular junction molecules. To further explore the molecular mechanisms by which endothelial Myosin IIA causes BBB abnormalities, we systematically examined the expression of key molecules regulating BBB integrity. It was observed that only the mRNA levels of the *Ctnnb1* gene were decreased, with a significant reduction in its protein levels as well. Previous research has demonstrated that the endothelial β-catenin signaling pathway is crucial for maintaining BBB integrity and central nervous system homeostasis in adult mice; mice with β-catenin deficiencies exhibit spontaneous epileptic symptoms, along with neurodegeneration, cerebral hemorrhage, and symptoms of central nervous system inflammation [52,54]. Interestingly, the locations of tracer leakage in the brain tissue of *Myh9*^ECKO^ mice were similar to those in mice with β-catenin deficiencies [71,72].

Our study confirmed that β-catenin acts as a key downstream molecule in the regulation of brain endothelial barrier function by Myosin IIA. It explored the possible mechanisms by which brain endothelial Myosin IIA regulates β-catenin, finding that the transcription level of the *Ctnnb1* gene in the *Myh9*^ECKO^ mouse brain endothelium was significantly reduced. The literature research revealed that, in a mouse model of gastric cancer, Myosin IIA in cancer cells can form complexes with other molecules and bind to the promoter region of the *Ctnnb1* gene to mediate its transcriptional expression [55]. Initially, we employed immunofluorescence staining to detect the expression of Myosin IIA in the nuclei of brain ECs, followed by constructing a luciferase reporter plasmid containing the *Ctnnb1* gene promoter fragment. The results from the dual-luciferase reporter system suggest that Myosin IIA in brain ECs can mediate the transcription of *Ctnnb1*, although it does not rule out the possibility that Myosin IIA could also influence β-catenin protein expression through other mechanisms. Furthermore, we demonstrated via co-immunoprecipitation that Myosin IIA can bind to the β-catenin protein. Future studies could involve cellular or tissue staining to determine the co-localization of Myosin IIA and β-catenin, and to identify their specific binding sites. Mutations at these binding sites could be explored to investigate their impact on the integrity of the BBB.

It is well known that tight junction proteins exhibit altered cellular trafficking patterns in response to pathological stressors. For example, studies have indicated that Occludin trafficking is altered in response to inflammatory and oxidative stress conditions [73,74]. In our study, we proposed that changes in the de novo synthesis of tight junction proteins are responsible for the altered paracellular permeability observed when Myosin IIA is knocked out. However, it is also possible that Myosin IIA abnormality may affect the trafficking of tight junction proteins to the plasma membrane, in addition to its effects on de novo synthesis. Along this line, it has been reported that Myosin IIA facilitates vesicle trafficking for MG53-mediated cell membrane repair [75]. Therefore, future research should investigate whether Myosin IIA interacts with other molecules involved in the trafficking of tight junction proteins and explore how these interactions may contribute to the maintenance of BBB integrity.

Myosin IIA is involved in numerous physiological processes, including cell adhesion, migration, and cytoskeletal regulation, which raises concerns about the safety of directly targeting this protein for improving drug delivery into the brain. While our findings indicate that Myosin IIA deficiency increases BBB permeability, allowing larger molecules to pass through, the broad function of Myosin IIA across various tissues suggests that targeting it could lead to off-target effects and undesirable consequences in other organs. Instead of directly inhibiting Myosin IIA, our data suggest that exploring specific downstream pathways or regulators associated with Myosin IIA may provide a safer approach. For example, modulating β-catenin signaling, which is regulated by Myosin IIA and plays a critical role in maintaining endothelial barrier function, could be a potential avenue for enhancing drug delivery while minimizing side effects. In conclusion, while Myosin IIA represents a novel target for BBB modulation, future studies should focus on more selective strategies that can harness its role in BBB permeability without compromising its systemic functions. This will be crucial for the development of safe and effective therapeutic interventions aimed at improving drug delivery to the brain.

## 5. Conclusions

In this study, we demonstrate that endothelial Myosin IIA is required for the maintenance of BBB integrity under physiological conditions. The deficiency of endothelial Myosin IIA in the brain exacerbates the pathogenesis of epilepsy. Mechanistically, Myosin IIA exerts the aforementioned effect through β-catenin. Overall, this study unveils new functions and the underlying mechanism of endothelial Myosin IIA, providing potential therapeutic targets for related diseases.

## Data Availability

The original contributions presented in the study are included in the article/Supplementary Material, further inquiries can be directed to the corresponding author.

## Supplementary Materials

The supporting information can be downloaded at: https://www.mdpi.com/article/10.3390/cells13191635/s1, Figure S1. Endothelial Myosin IIA controls BBB integrity. Figure S2. Normal brain vascular structure in *Myh9*^ECKO^ mice. Figure S3. Induced endothelial-specific knockout of Myh9 disrupts the BBB integrity. Figure S4. Deletion of brain endothelial Myosin IIA downregulates junctional proteins of the BBB. Figure S5. Endothelial Myosin IIA deficiency does not affect GLUT1 expression. Figure S6. *Myh9**ECKO* mice show intact basement membranes, pericyte coverage, and astrocyte adhesion. Figure S7. Overexpression of β-catenin improves the protein expression levels of junctional molecules caused by Myosin IIA deficiency. *Myh9*^ECKO^ mice show intact basement membranes, pericyte coverage, and astrocyte adhesion. Table S1. Information regarding the antibodies used for IHC and FACS. Table S2. Information regarding the antibodies used for Western blot. Table S3. Primers for real-time PCR analysis.
