# Supplementary material for: Endothelial Myosin IIA Is Required for the Maintenance of Blood–Brain Barrier Integrity
Source: Cells. 2024 Oct 1;13(19):1635. doi: 10.3390/cells13191635 (PMC11475711; doi:10.3390/cells13191635)
Supplement: Supplementary file 1 [file cells-13-01635-s001.zip › Deng et al Supplementary Materials.pdf]

Supplementary Materials

Endothelial Myosin IIA is required for the maintenance of blood-brain barrier integrity

Yanan Deng[1], Ziqi Qiao[1], Changping Zhou[1], Yujun Pei[1], Han Xu[1], Xuya Kang[1], Jincai Luo[1]*

[1]Institute of Molecular Medicine, School of Future Technology, Beijing Key Laboratory of Cardiometabolic Molecular Medicine, Peking University, Beijing 100871, China.

***Corresponding Authors:**

Prof. Jincai Luo, Institute of Molecular Medicine/College of Future Technology, Peking University, Beijing 100871, China. E-mail: jincailuo@pku.edu.cn.

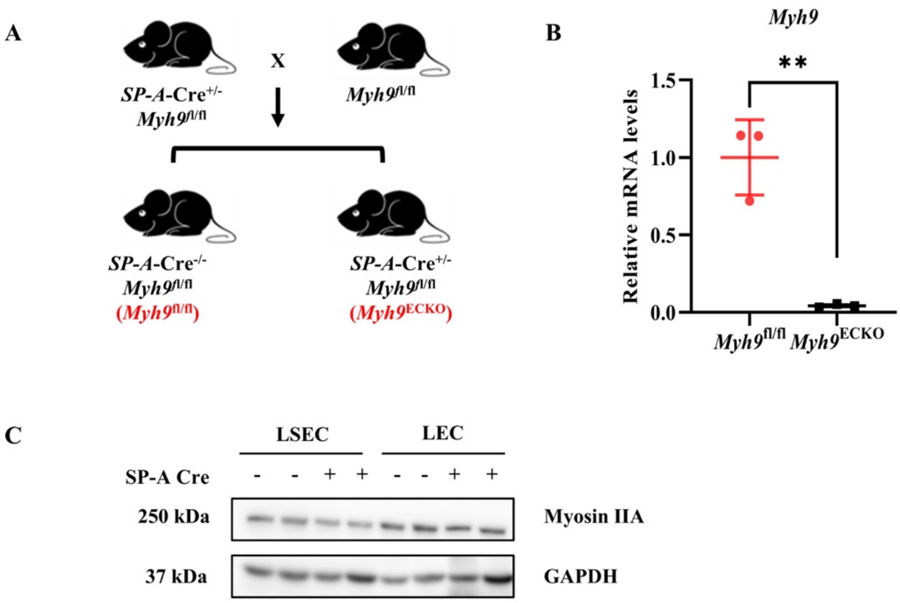

D

Breeding: *SP-A*-Cre$^{+/-}$ *Myh9*$^{fl/fl}$ X *SP-A*-Cre$^{-/-}$ *Myh9*$^{fl/fl}$

P0	*SP-A*-Cre$^{+/-}$ *Myh9*$^{fl/fl}$	*SP-A*-Cre$^{-/-}$ *Myh9*$^{fl/fl}$	Total (n)
Observed	274	287	562
Expected	281	281	562

Figure S1. Endothelial Myosin IIA controls BBB integrity

A. Breeding strategy for *Myh9*^{ECKO} mice.

B. RT-PCR analysis of *Myh9* mRNA levels in primary brain ECs from *Myh9*$^{fl/fl}$ and *Myh9*^{ECKO} mice (n=3). ***p*<0.01; Student's t-test.

C. Representative western blot image showing Myosin IIA protein levels in LSECs and LECs from *Myh9*$^{fl/fl}$ and *Myh9*^{ECKO} mice.

D. Statistical analysis of offspring numbers from different genotypes in *Myh9*^{ECKO} mice.

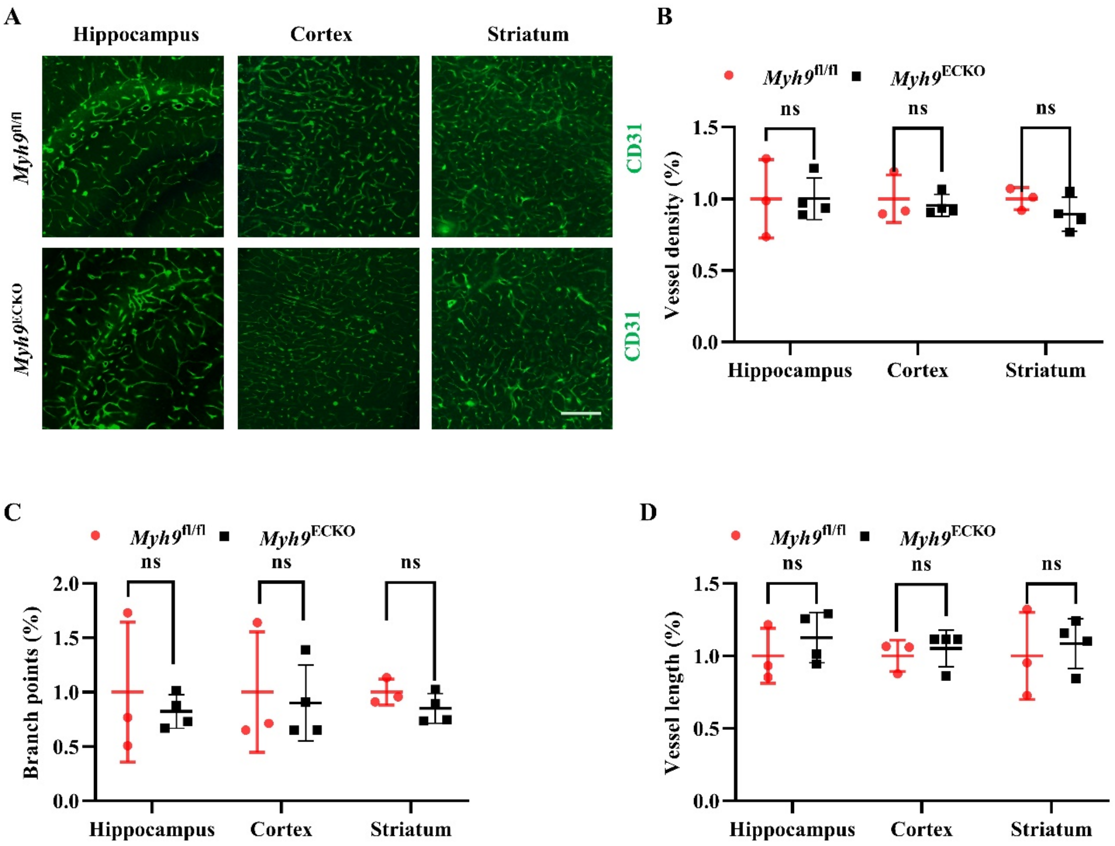

Figure S2. Normal brain vascular structure in *Myh9*^{ECKO} mice

A. Representative confocal microscopy images of vascular morphology in different brain regions of *Myh9*$^{\mathrm{fl/fl}}$ and *Myh9*^{ECKO} mice, stained for CD31 (green). Regions include the hippocampus, cortex, and striatum. Scale bar: 200 μm.

B. Statistical analysis of vascular density in different brain regions of *Myh9*$^{\mathrm{fl/fl}}$ and *Myh9*^{ECKO} mice (*Myh9*$^{\mathrm{fl/fl}}$, n=3; *Myh9*^{ECKO}, n=4). ns, $p>0.05$; Student's t-test.

C. Statistical analysis of vascular branching in different brain regions of *Myh9*$^{\mathrm{fl/fl}}$ and *Myh9*^{ECKO} mice (*Myh9*$^{\mathrm{fl/fl}}$, n=3; *Myh9*^{ECKO}, n=4). ns, $p>0.05$; Student's t-test.

D. Statistical analysis of vascular length in different brain regions of *Myh9*$^{\mathrm{fl/fl}}$ and *Myh9*^{ECKO} mice (*Myh9*$^{\mathrm{fl/fl}}$, n=3; *Myh9*^{ECKO}, n=4). ns, $p>0.05$; Student's t-test.

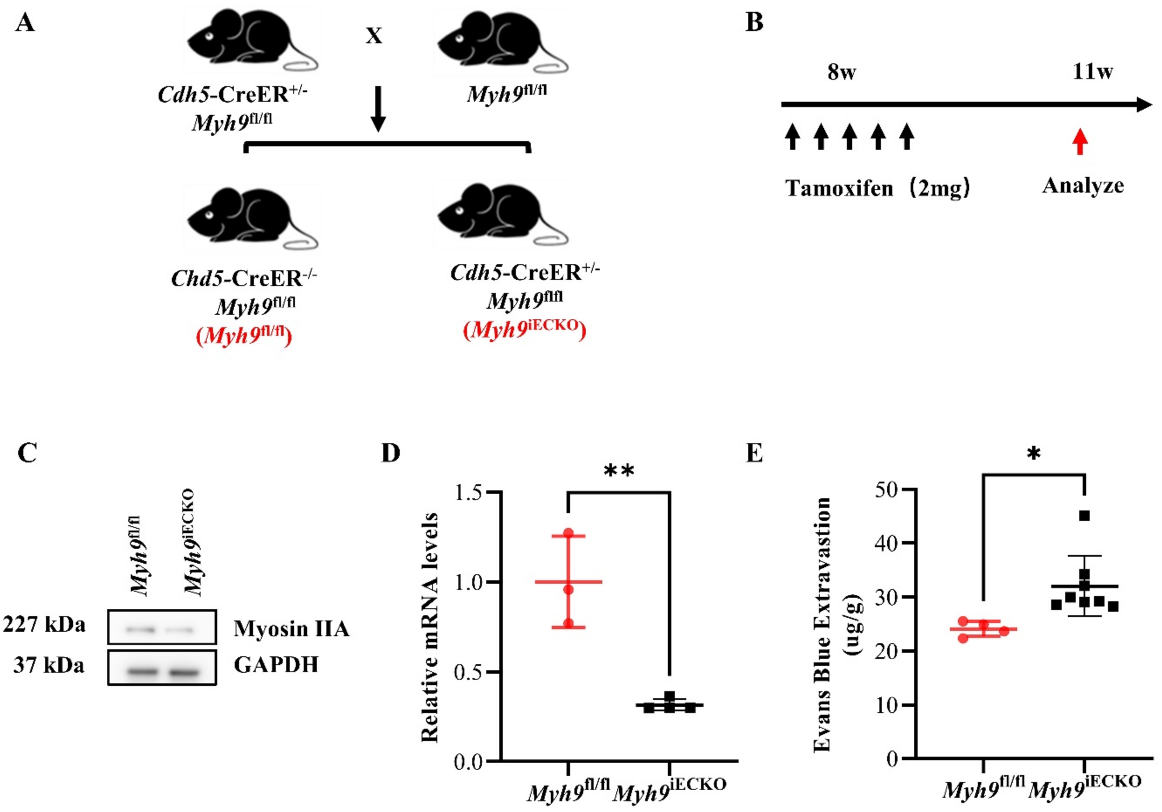

Figure S3. Induced endothelial-specific knockout of *Myh9* disrupts the BBB integrity

A. Breeding strategy for *Myh9*^{iECKO} mice.

B. Induction strategy for *Myh9*^{iECKO} mice.

C. Western blot analysis of Myosin IIA protein levels in primary brain ECs from *Myh9*$^{\text{fl/fl}}$ and *Myh9*^{iECKO} mice.

D. RT-PCR analysis of *Myh9* mRNA levels in primary brain ECs from *Myh9*$^{\text{fl/fl}}$ and *Myh9*^{iECKO} mice (*Myh9*$^{\text{fl/fl}}$, n=3; *Myh9*^{iECKO}, n=4). **p<0.01; Student's t-test.

E. Statistical analysis of Evans Blue tracer leakage into the brain parenchyma of *Myh9*$^{\text{fl/fl}}$ and *Myh9*^{iECKO} mice following intravenous injection (*Myh9*$^{\text{fl/fl}}$, n=4; *Myh9*^{iECKO}, n=8). *p<0.05; Student's t-test.

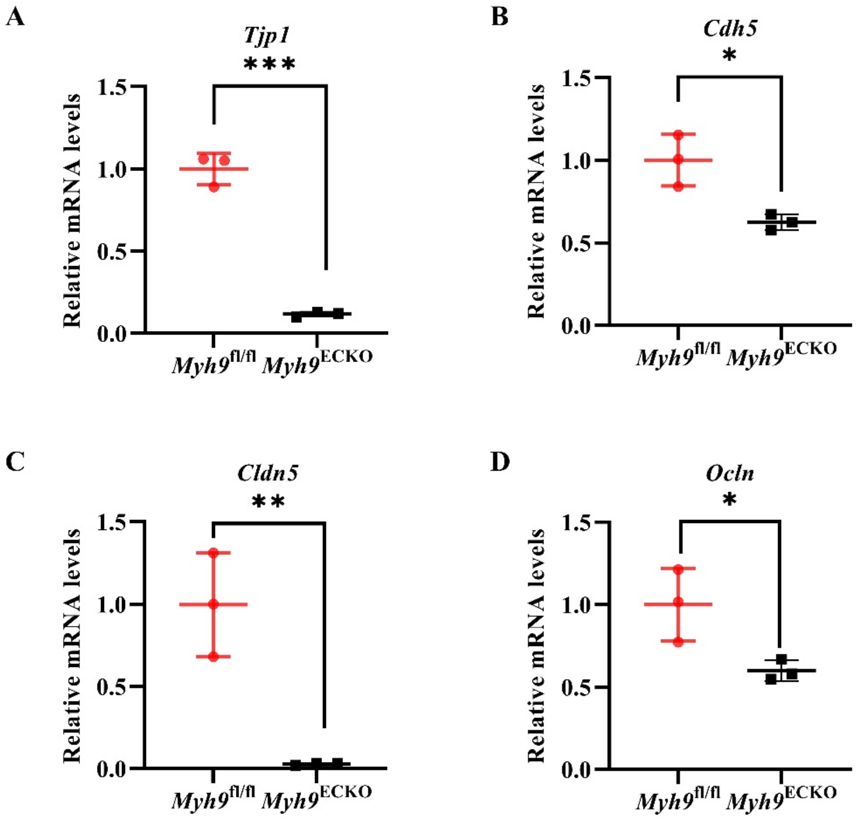

Figure S4. Deletion of brain endothelial Myosin IIA downregulates junctional proteins of the BBB

A. RT-PCR analysis of *Tjp1* mRNA levels in primary brain ECs from *Myh9*$^{fl/fl}$ and *Myh9*^{ECKO} mice (n=3). ***p<0.001; Student's t-test.

B. RT-PCR analysis of *Cdh5* mRNA levels in primary brain ECs from *Myh9*$^{fl/fl}$ and *Myh9*^{ECKO} mice (n=3). *p<0.05; Student's t-test.

C. RT-PCR analysis of *Ocln* mRNA levels in primary brain ECs from *Myh9*$^{fl/fl}$ and *Myh9*^{ECKO} mice (n=3). **p<0.01; Student's t-test.

D. RT-PCR analysis of *Cldn5* mRNA levels in primary brain ECs from *Myh9*$^{fl/fl}$ and *Myh9*^{ECKO} mice (n=3). *p<0.05; Student's t-test.

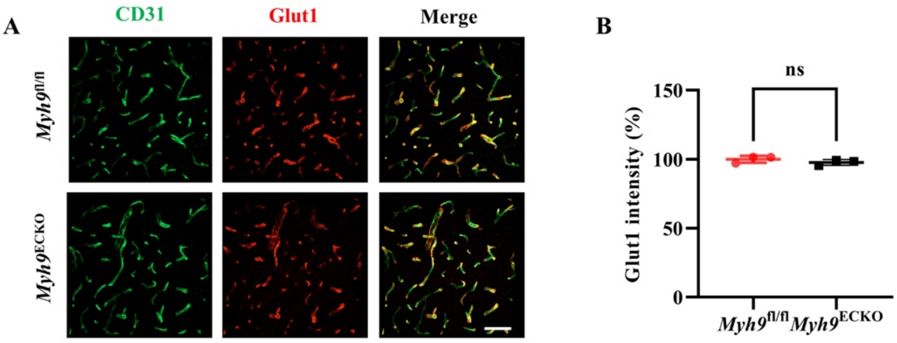

Figure S5. Endothelial Myosin IIA deficiency does not affect GLUT1 expression

A. Representative immunofluorescence co-staining images of brain sections from *Myh9*$^{fl/fl}$ and *Myh9*^{ECKO} mice showing CD31 (green) and Glut1 (red). Scale bar: 120 µm.

B. Statistical analysis of colocalization between Glut1 and CD31 in brain sections from *Myh9*$^{fl/fl}$ and *Myh9*^{ECKO} mice (n=3). ns, p>0.05; Student's t-test.

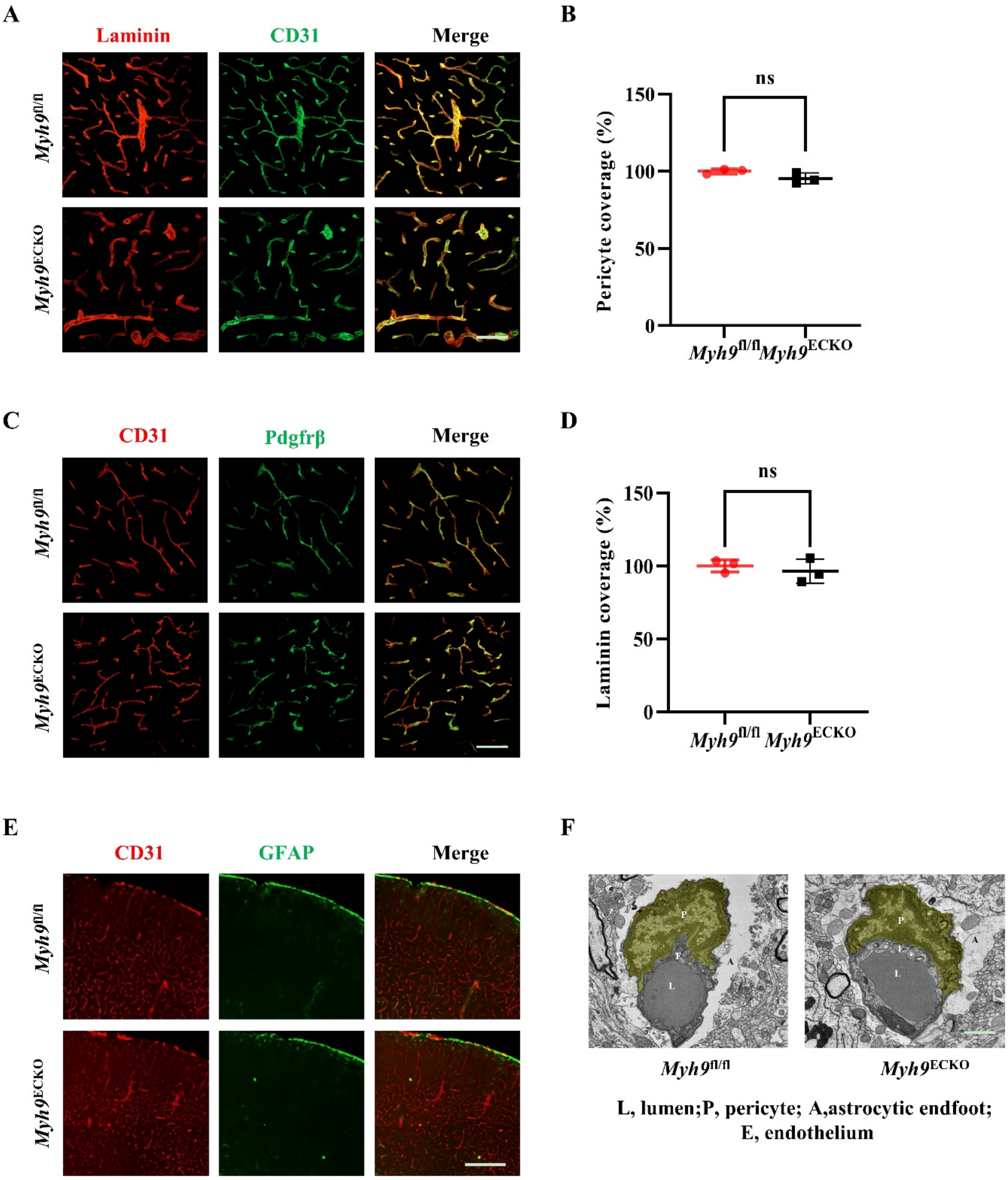
A
Laminin
CD31
Merge
Myh9fl/fl
Myh9ECKO
B
ns
Pericyte coverage (%)
150
100
50
0
Myh9fl/fl
Myh9ECKO
C
CD31
Pdgfrβ
Merge
Myh9fl/fl
Myh9ECKO
D
ns
Laminin coverage (%)
150
100
50
0
Myh9fl/fl
Myh9ECKO
E
CD31
GFAP
Merge
Myh9fl/fl
Myh9ECKO
F
Myh9fl/fl
Myh9ECKO
L, lumen;P, pericyte; A,astrocytic endfoot;
E, endothelium

Figure S6. *Myh9*^{ECKO} mice show intact basement membranes, pericyte coverage, and astrocyte adhesion

A. Representative immunofluorescence co-staining images of brain sections from *Myh9*$^{fl/fl}$ and *Myh9*^{ECKO} mice, showing the basement membrane marker Laminin (red) and endothelial marker CD31 (green). Scale bar: 100 μm.

B. Statistical analysis of colocalization between Laminin and CD31 in brain sections from *Myh9*$^{fl/fl}$ and *Myh9*^{ECKO} mice (n=3). ns, p>0.05; Student's t-test.

C. Representative immunofluorescence co-staining images of brain sections from *Myh9*$^{fl/fl}$ and *Myh9*^{ECKO} mice, showing CD31 (red) and pericyte marker Pdgfr β (green). Scale bar: 120 μm.

D. Statistical analysis of colocalization between CD31 and Pdgfr β in brain sections from *Myh9*$^{fl/fl}$ and *Myh9*^{ECKO} mice (n=3). ns, p>0.05; Student's t-test.

E. Representative immunofluorescence co-staining images of brain sections from *Myh9*$^{fl/fl}$ and *Myh9*^{ECKO} mice, showing astrocyte marker CD31 (red) and GFAP (green). Scale bar: 300 μm.

F. Representative electron microscopy images of the blood-brain barrier structure in *Myh9*$^{fl/fl}$ and *Myh9*^{ECKO} mice, showing the vascular lumen, pericytes, astrocyte endfeet, and endothelium. Scale bar: 2 μm.

A

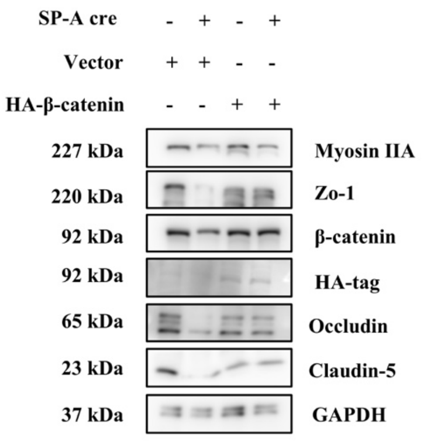

Figure S7. Overexpression of β-catenin improves the protein expression levels of junctional molecules caused by Myosin IIA deficiency

A. Changes in protein expression levels of cell junction molecules were assessed by western blot of brain ECs from *Myh9*$^{fl/fl}$ and *Myh9*^{ECKO} mice after being injected with AAV-GFP virus and AAV-HA-β-catenin virus.

S

Table S1. Information regarding the antibodies used for IHC and FACS

Antibody	Host	Dilution	Resource	Catalog
Claudin-5	Rabbit	1:1000	Invitrogen	341600
Occludin	Rabbit	1:1000	Invitrogen	711500
ZO-1	Rabbit	1:1000	Invitrogen	402200
Myosin IIA	Rabbit	1:1000	Proteintech	14844-1-AP
β-catenin	Rabbit	1:1000	CST	8480T
CD31	Goat	1:1000	RD	AF3628
GFAP	Rabbit	1:500	Sigma	AB5804
Pdgfrβ	Rat	1:1000	Invitrogen	14-1402-82
Laminin	Rabbit	1:1000	Sigma	L9393
GLUT1	Rabbit	1:1000	Abcam	ab652
FITC-CD31	Rat	1:300	eBiosciences	11-0311-85
Per-cp-CD45	Mouse	1:300	eBiosciences	45-0415-82
PE-Pdgfrβ	Mouse	1:300	eBiosciences	12-1402-81

Table S2. Information regarding the antibodies used for western blot

Antibody	Host	Dilution	Resource	Catalog
Myosin IIA	Rabbit	1:1000	Sigma	M8064
Claudin-5	Rabbit	1:1000	Invitrogen	341600
Occludin	Rabbit	1:1000	Invitrogen	711500
ZO-1	Rabbit	1:1000	Invitrogen	402200
β-catenin	Rabbit	1:1000	CST	8480T
VE-cadherin	Rat	1:1000	Invitrogen	14-1441-82
GAPDH	Rabbit	1:1000	CST	5174T
HRP-anti-Rabbit	Goat	1:3000	GE	NA9340
HRP-anti-Rat	Goat	1:3000	Easybio	BE0108
HRP-anti-Mouse	Goat	1:3000	GE	NA9310

Table S3. Primers for real-time PCR analysis

Primer	5' to 3' (sequences)
Myh9 F	CGCTCGAGAAAGTCCACTCG
Myh9 R	GGCCAGCGGGTTATTGATGA
GAPDH F	AGGTCGGTGTGAACGGATTTG
GAPDH R	TGTAGACCATGTAGTTGAGGTCA
Tjp1 F	GATTTACCCGTCAGCCCTTCT
Tjp1 R	TCGCAAACCCACACTATCTCC
Ocln F	AGATTCCTCTGACCTTGAGTGTGG
Ocln R	TCCTGCTTTCCCCTTCGTG
Cdh5 F	TAGCAAGAGTGCGCTGGAGATTCA
Cdh5 R	ACACATCATAGCTGGTGGTGTCCA
Cldn5 F	GCAAGGTGTATGAATCTGTGCT
Cldn5 R	GTCAAGGTAACAAAGAGTGCCA
Pecam1 F	TTGAGCCTCACCAAGAGAACGGAA
Pecam1 R	AATCCAGGAATCGGCTGCTCTTCT
Tek F	AGGAAGAAAAGCGAGGGAAATG
Tek R	CAGGTGAGGAGCAGAGTTGAGAG
S1pr1 F	ATGGTGTCCACTAGCATCCC
S1pr1 R	CGATGTTCAACTTGCCTGTGTAG
Unc5b F	GCTGCCGTACTTCCTATTGGA
Unc5b R	CCCACTCGCCATTACACTTGA
Lsr F	CTACAACCCCTATGTGGAGTGC
Lsr R	CTGCCCTGGTAGTAGTCTCCC
JAM F	ACAAGGCAAGGGTTCGGTG
JAM R	CAGGTCAATTTGATGGACTCGTT
Slc2a1 F	CAGTTCGGCTATAACACTGGTG
Slc2a1 R	GCCCCCGACAGAGAAGATG
Plvap F	GACTACGCGACGTGAGATGGA
Plvap R	AGGATGATAGCGGCGATGAAG
MCT1 F	ACCTACGTTGGGTTCTGTGTC
MCT1 R	TGAGGATCACGCCACAAGC

Table S3. Primers for real-time PCR analysis

Primer	5' to 3' (sequences)
Mfsd2a F	CGTGCGGGAGCAGAGAGA
Mfsd2a R	AAGCCAGGGAGGTAAAAAGGAAG
*Ctnnb*1 F	ATGGAGCCGGACAGAAAAGC
Ctnnb1 R	CTTGCCACTCAGGGAAGGA
