# Supplementary material for: Endothelial Myosin IIA Is Required for the Maintenance of Blood–Brain Barrier Integrity
Source: Cells. 2024 Oct 1;13(19):1635. doi: 10.3390/cells13191635 (PMC11475711; doi:10.3390/cells13191635)

Original Images for Blots/Gels

Figure 1A

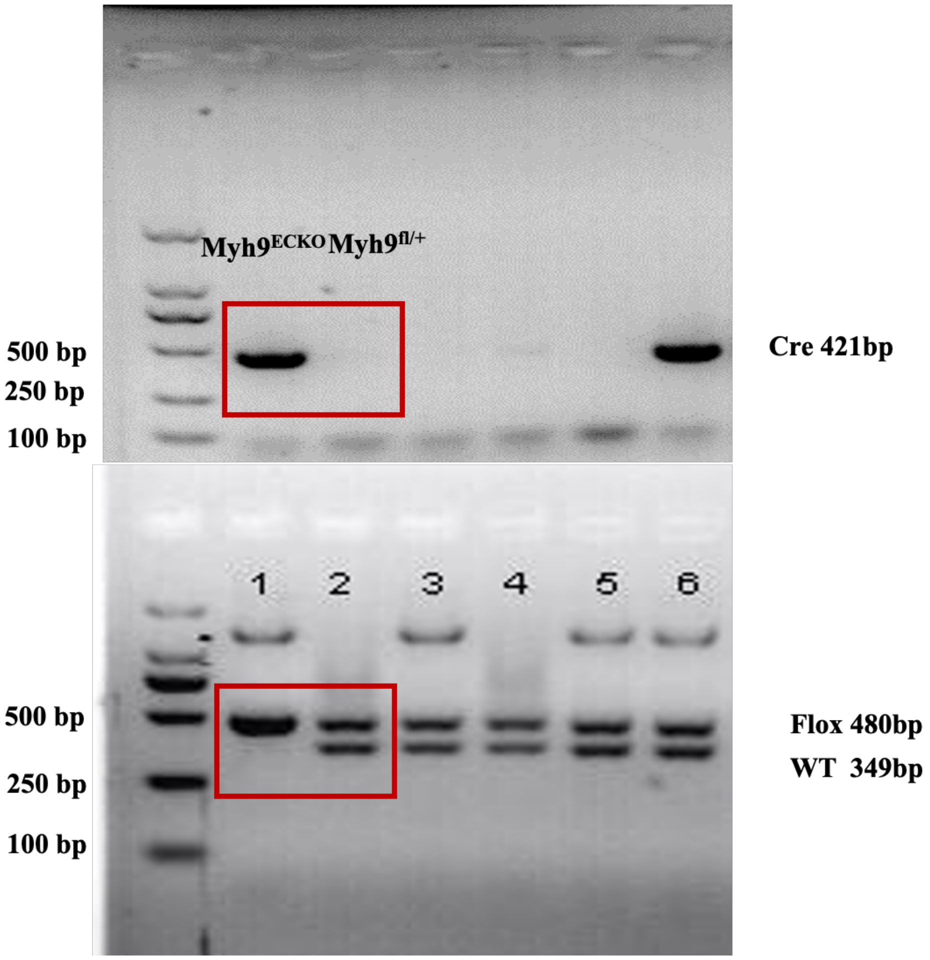

Figure 1B

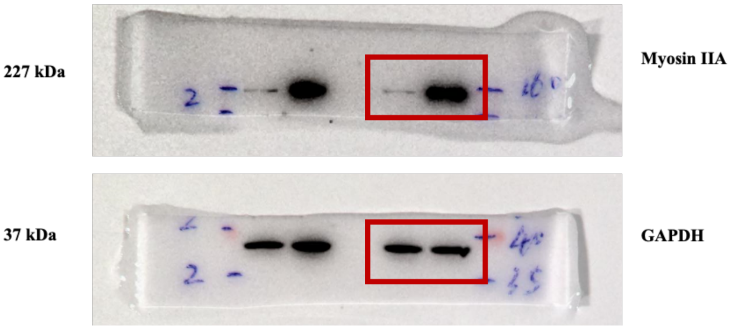

Figure 3C

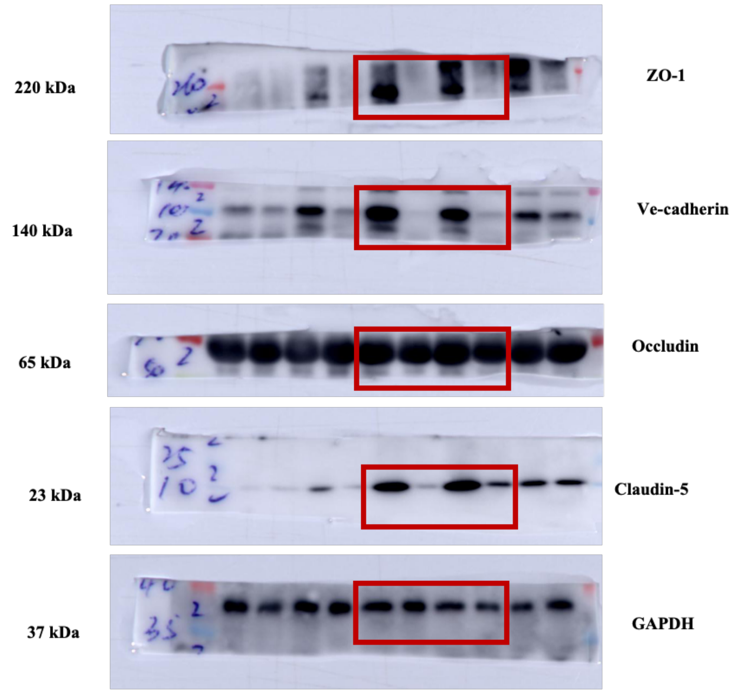

Figure 4B

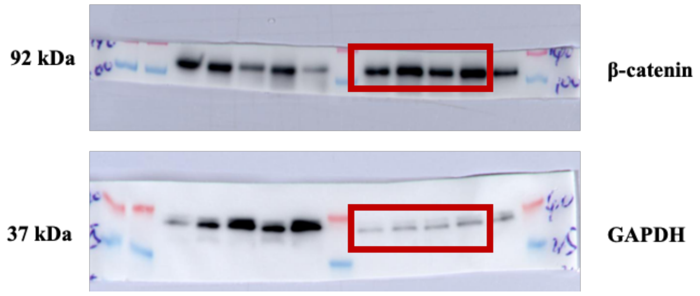

Figure 4I

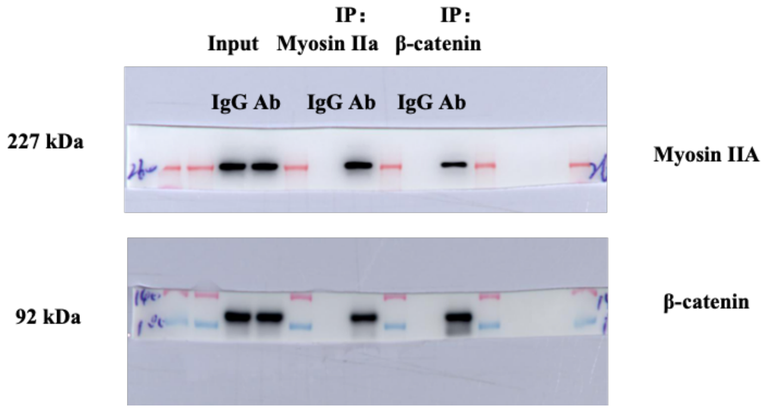

Figure 5B

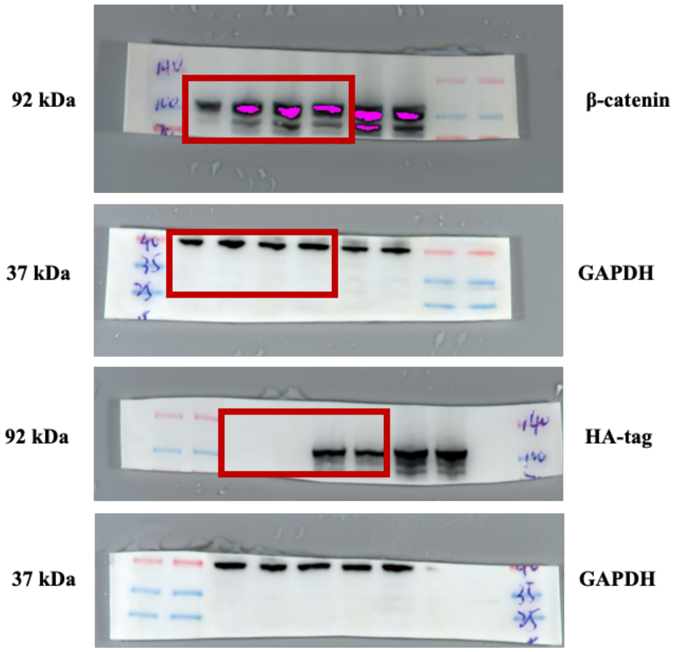

Figure S1C

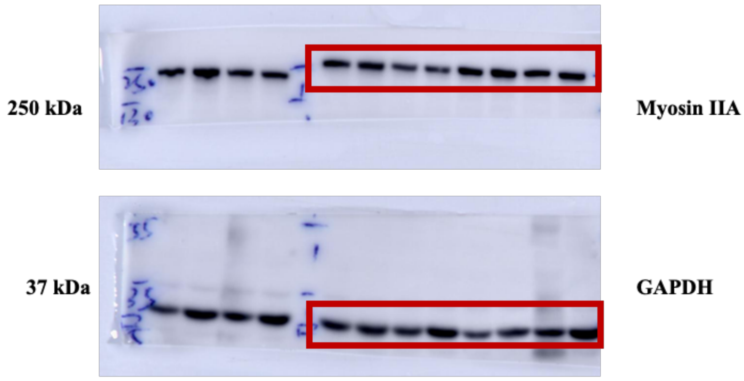

Figure S3C

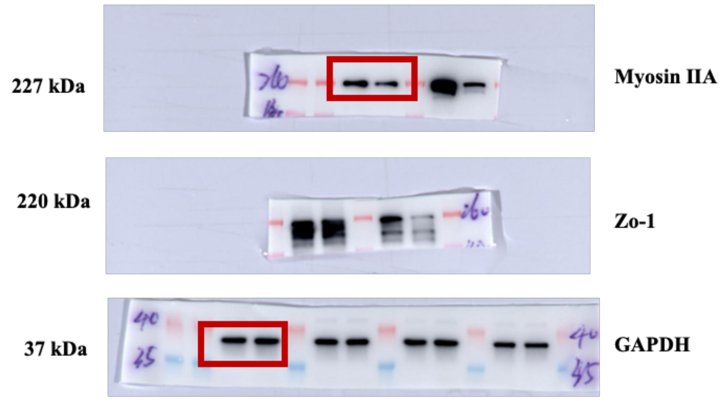

Figure S7A

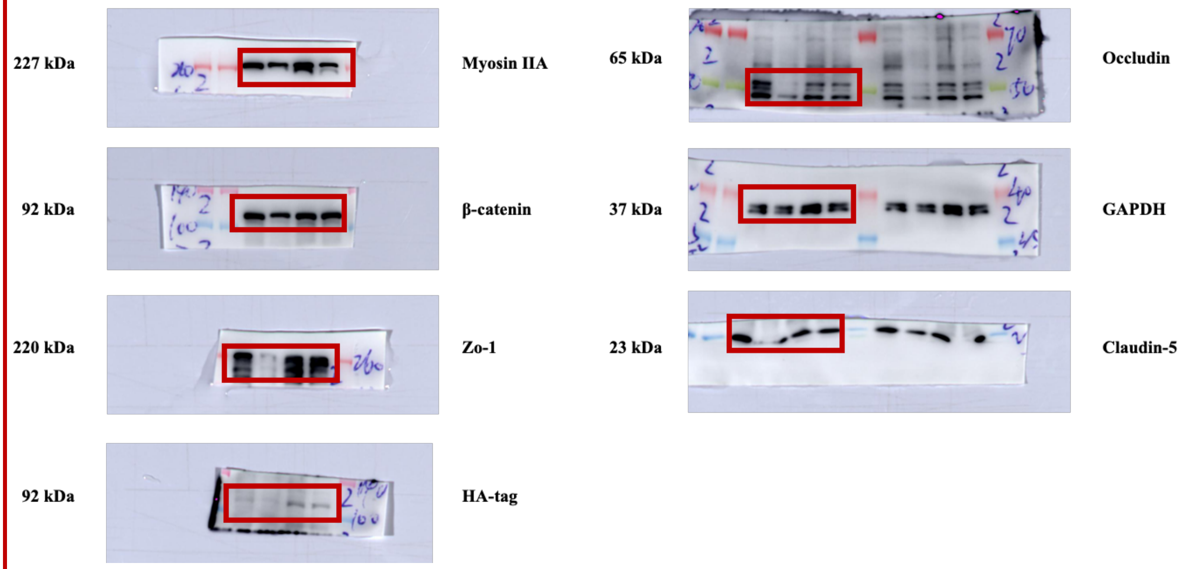

Supplement: Supplementary file 1 [file cells-13-01635-s001.zip › Deng et al-Original Images for Blots.pdf]
